# Obtaining control of cell surface functionalizations via Pre-targeting and Supramolecular host guest interactions

**DOI:** 10.1038/srep39908

**Published:** 2017-01-06

**Authors:** Mark T. M. Rood, Silvia J. Spa, Mick M. Welling, Jan Bart ten Hove, Danny M. van Willigen, Tessa Buckle, Aldrik H. Velders, Fijs W. B. van Leeuwen

**Affiliations:** 1 Interventional Molecular Imaging Laboratory, Department of Radiology, Leiden University Medical Center, Albinusdreef 2, PO BOX 9600, 2300 RC, Leiden, The Netherlands; 2 Laboratory of BioNanoTechnology, Axis, Building 118, Bornse weilanden 9, 6708 WG Wageningen, The Netherlands

## Abstract

The use of mammalian cells for therapeutic applications is finding its way into modern medicine. However, modification or “training” of cells to make them suitable for a specific application remains complex. By envisioning a chemical toolbox that enables specific, but straight-forward and generic cellular functionalization, we investigated how membrane-receptor (pre)targeting could be combined with supramolecular host-guest interactions based on β-cyclodextrin (CD) and adamantane (Ad). The feasibility of this approach was studied in cells with membranous overexpression of the chemokine receptor 4 (CXCR4). By combining specific targeting of CXCR4, using an adamantane (Ad)-functionalized Ac-TZ14011 peptide (guest; K_D_ = 56 nM), with multivalent host molecules that entailed fluorescent β-CD-Poly(isobutylene-*alt*-maleic-anhydride)-polymers with different fluorescent colors and number of functionalities, host-guest cell-surface modifications could be studied in detail. A second set of Ad-functionalized entities enabled introduction of additional surface functionalities. In addition, the attraction between CD and Ad could be used to drive cell-cell interactions. Combined we have shown that supramolecular interactions, that are based on specific targeting of an overexpressed membrane-receptor, allow specific and stable, yet reversible, surface functionalization of viable cells and how this approach can be used to influence the interaction between cells and their surroundings.

Cells are the cornerstones of mammalian life forms, their versatile surfaces are naturally evolved to express molecules which provide a refined means to interact with their surroundings, e.g. functionalizations^1^. These functionalities can stimulate and/or respond to cellular activity^2^, regulate vital processes such as hormone balance^3^,^4^ and induce immune responses^5^,^6^. The strength of using cells for therapeutic purposes is progressively being recognized in medicine and is (among others) exploited in the form of immunotherapy and (stem) cell transplantations^7^,^8^,^9^. The implantation of (stem) cells allows regeneration of the impaired tissue, however therapy efficiency is limited by low engraftment of the cells at the site of interest^10^. Furthermore, such cell-based therapies often go hand-in-hand with relatively complex biological modification processes such as genetic modification^11^ or metabolic labeling^12^. As each medical application desires specialized features and functions, these modification processes match an individual cell type to a specific application. To enhance delivery and local retention to the site of interest, ideally an interaction-enhancing functionalization can be introduced in a cell-type specific manner, using well defined and generic (chemical) functionalization approaches^13^.

By recognizing the cell surface as a (complex) chemical scaffold, one can reason that its functionality and interactions can be altered via bio-orthogonal conjugations^14^,^15^,^16^. Known examples are the introduction of polyelectrolyte polymers^14^,^17^,^18^,^19^ and the insertion of lipophilic anchors containing a functional group such as integrins or reactive handles^20^,^21^,^22^,^23^,^24^. Alternatively, one could approach cell functionalization in a way similar to the functionalization of inorganic surfaces. The opposite has been used extensively; hereby inorganic surfaces with simulated cell surfaces have been applied to mimic interactions that occur in nature^25^,^26^,^27^. When controllable and reversible inorganic-surface modifications are desired in aqueous environments, supramolecular host-guest interactions, e.g. using beta-cyclodextrin (β-CD) and adamantane (Ad), provide outcome^28^,^29^,^30^,^31^,^32^,^33^. Especially when one considers that CD based host-guest interactions also play a key role in the preparation of biomedical materials and in drug delivery^33^,^34^,^35^.

Membrane-expressed biomarkers provide a unique fingerprint for cell populations and allow efficient and specific targeting using vectors such as antibody-derivatives and peptides^36^. Such vectors are routinely used for applications in imaging and therapy^37^. Not only can specificity be achieved by direct targeting of the receptor, indirect targeting can also be applied in a pre-targeting setup^38^. Here a receptor-targeting vector is first directed towards the membrane-receptor. This first targeting step is then followed by a secondary functionalization, using an agent that contains e.g. a diagnostic or a therapeutic label^38^,^39^,^40^. Other than applying the pre-targeting concept to introduce such diagnostic/therapeutic labels, potentially the same concept could also be utilized to introduce other functionalities on the cell surfaces.

We reasoned that it would be possible to functionalize cell surfaces in a similar way to what is known for inorganic surfaces. To realize this, a combination of membrane receptor-(pre)targeting and supramolecular surface functionalization techniques were used. Herein the chemokine receptor 4 (CXCR4)^41^, a receptor that plays a key role in cellular motility as result of chemotaxis, served as the membrane receptor. Specific functionalization of CXCR4 was achieved via the use of an adamantane functionalized Ac-TZ14011 peptide (Fig. 1a1). Further surface functionalization was based on the host-guest interaction between beta-cyclodextrin host molecules on fluorescent beta-cyclodextrin-Poly(isobutylene-*alt*-maleic-anhydride)-polymers and the adamantane functionality (Fig. 1a2). We also illustrate how such an approach enables the introduction of additional surface functionalities (e.g. diagnostic labels) and can even be used to drive cell-cell interactions.

## Results and Discussion

### Design and synthesis of the chemical components

**Figure 1:**
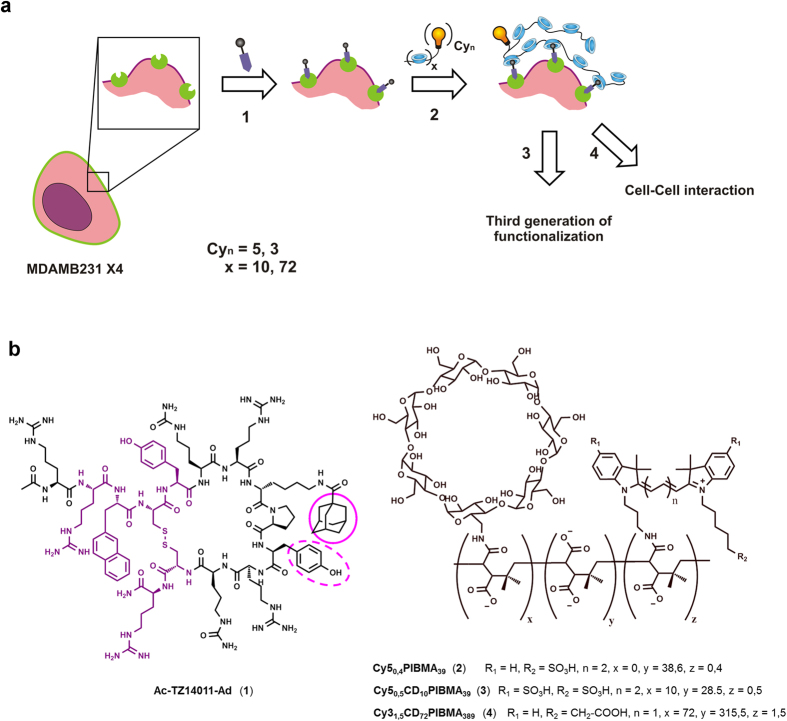
(**a**) Schematic representation of the supramolecular functionalization of cell surfaces via targeting of the membrane-receptor CXCR4 (green). As first step, cellular specificity is introduced by using **Ac-TZ14011-Ad** (**1**) peptide to target CXCR4 (step **1**). This provides an Ad-functionality on the surface that can be used as basis for more generic functionalization with β-CD polymers containing variable fluorescent labels and β-CD; **Cy5**_**0.5**_**CD**_**10**_**PIBMA**_**39**_(**3**). **Cy3**_**1.5**_**CD**_**72**_**PIBMA**_**389**_ (**4**) (step **2**; x = 10 or 72). The then artificially generated CD-surfaces can be used to drive cellular interactions with entities containing matching guest functionalities. Hereby a third generation of functionalization can be introduced such as Ad-functionalized fluorescent dye (step **3**) or cell-cell interactions can be induced with Ad-functionalized cells (step **4**). (**b)** Chemical structures of the key compounds; **Ac-TZ14011-Ad** (**1**), **Cy5**_**0.5**_**CD**_**10**_**PIBMA**_**39**_(**3**), **Cy3**_**1.5**_**CD**_**72**_**PIBMA**_**389**_ (**4**) and **Cy5**_**0.4**_**PIBMA**_**39**_(**2**), the polymer-units containing different functionalities are randomly distributed within the polymer. In **Ac-TZ14011-Ad** the pharmacophore of Ac-TZ14011 is indicated in purple. The main guest for β-CD; Ad is indicated by a pink solid line, together with a possible second guest: Tyr10 (pink dotted line).

The cyclic Ac-TZ14011 peptide, a well-known targeting ligand for the CXCR4 receptor^41^, was functionalized using an Ad-group (Fig. 1b). Hereby the Ad-group pointed outwards from the pharmacophore^42^, making the Ad-group available for interactions with the cell’s environment. Flow cytometry-based competition experiments on viable CXCR4 expressing cells (MDAMB231 × 4), revealed a K_D_ of 56 nM for **Ac-TZ14011-Ad** (Supplementary Fig. S11), using the fluorescent Ac-TZ14011-MSAP (K_D_ = 187 nM) as a reference. Unmodified Ac-TZ14011 has an affinity of 8.6 nM^43^, which indicates that the introduction of the Ad functionality only has a relatively small adverse effect on the receptor affinity^41^.

Poly(isobutylene-*alt*-maleic-anhydride) (PIBMA) with different lengths (PIBMA_39_ and PIBMA_389_) were used for the polymer backbone, as the anhydrides allow easy grafting with nucleophiles such as β-CD-NH_2_, Cy3-NH_2_ and Cy5-NH_2_. Furthermore, hydrolyzing the non-reacted anhydrides to carboxylates, provides good solubility in aqueous solutions (pKa_1_ = 4)^44^,^45^. This approach resulted in the synthesis of two fluorescent β-CD-PIBMA-polymers and one solely fluorescent PIBMA_39_-polymer without β-CD for control experiments. After conjugation, absorption spectroscopy revealed that on average 0.5 Cy5, 1.5 Cy3, and 0.4 Cy5 fluorophores were conjugated to the respective polymers. ^1^H-NMR and NMR Diffusion Ordered Spectroscopy (DOSY) were used to determine the degree of CD-functionalization and to estimate the hydrodynamic diameter of the respective polymers. This yielded one polymer with 10 β-CD-units with diameter ~2.8 nm (~18.8 kDa: **Cy5**_**0.5**_**CD**_**10**_**PIBMA**_**39**_), one polymer with 72 β-CD-units with diameter ~11.7 nm (~155 kDa; **Cy3**_**1.5**_**CD**_**72**_**PIBMA**_**389**_), and one polymer without β-CD-units with diameter ~2.7 nm (~7.8 kDa; **Cy5**_**0.4**_**PIBMA**_**39**_)(Fig. 1b).

### Functionalization of cell surfaces

**Figure 2:**
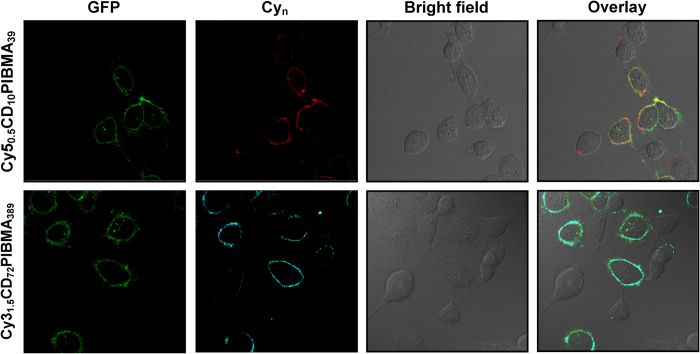
Supramolecular surface modification of viable MDAMB231 × 4 (with CXCR4-linked GFP-Tag) and MDAMB231 cells (without GFP-Tag) in mixed cell culture. Modification was accomplished via specific functionalization of the CXCR4 receptor with **Ac-TZ14011-Ad**, followed by host-guest interaction between β-CD molecules on fluorescent **Cy5**_**0.5**_**CD**_**10**_**PIBMA**_**39**_ or **Cy3**_**1.5**_**CD**_**72**_**PIBMA**_**389**_ polymers and the Ad functionality. Functionalization mainly occurs on the CXCR4 overexpressing MDAMB231 × 4 cells. For clarity, both the (overlay) image and the same image at the individual channels are displayed, with GFP in green, Cy5 (**Cy5**_**0.5**_**CD**_**10**_**PIBMA**_**39**_) in red, and Cy3 (**Cy3**_**1.5**_**CD**_**72**_**PIBMA**_**389**_) in blue.

To prove that (supramolecular) cell-surface modification becomes possible via specific functionalization of the membrane-receptors, CXCR4 overexpressing MDAMB231 × 4 cells were functionalized in two steps; first with **Ac-TZ14011-Ad** (1 h; 0 °C), to allow for CXCR4 receptor targeting (Fig. 1,1) and secondly with either **Cy5**_**0.5**_**CD**_**10**_**PIBMA**_**39**_or **Cy3**_**1.5**_**CD**_**72**_**PIBMA**_**389**_ (1 h; 0 °C) to allow further surface functionalization (Fig. 1,2). Cell analysis using confocal microscopy indicated that cell functionalization was accomplished using both polymer types (Supplementary Fig. S12). MTT cell-viability tests performed 24 h after the (supramolecular) cell-surface functionalization (Supplementary Fig. S13) showed that the cells were not negatively affected by the functionalization with **Ac-TZ14011-Ad** and either one of the two polymers (0–16 μM β-CD).

To further study the CXCR4-receptor specificity of the functionalization process, the experiments were repeated with a mixed cell culture of viable MDAMB231 × 4 (with overexpressed CXCR4 receptor and with CXCR4-linked GFP-tag) and as a control MDAMB231 cells (with basal CXCR4 expression and without CXCR4-linked GFP-tag). We have demonstrated previously that a fluorescent variant of the Ac-TZ14011 peptide allows for differentiation between the two cell lines, using their difference in CXCR4 expression levels^46^. Confocal microscopy (Fig. 2) and intensity analysis revealed that the average signal intensities of **Cy5**_**0.5**_**CD**_**10**_**PIBMA**_**39**_ and **Cy3**_**1.5**_**CD**_**72**_**PIBMA**_**389**_ were respectively 5 and 8 times higher on the MDAMB231 × 4 cells, compared to the signal intensities observed on the cells with basal CXCR4 expression (MDAMB231), which indicates receptor specificity.

**Figure 3:**
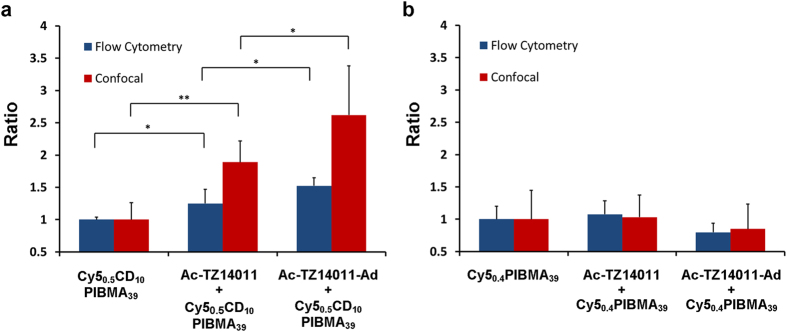
Host-guest interaction dependent cellular functionalization: (**a**) Binding of **Cy5**_**0.5**_**CD**_**10**_**PIBMA**_**39**_increases in a statistically significant manner when the guest moieties **Ac-TZ14011** and **Ac-TZ14011-Ad** become available at the cell surface. (**b**) **Cy5**_**0.4**_**PIBMA**_**39**_ functionalization is not influenced by the availability of guest moieties. These values remain at baseline. The degree of functionalization was quantified by Flow cytometry (blue) or Confocal microscopy (red). Graphs show the normalized data with the error bars indicating the standard deviations (n = 6) and the significance of differences marked with *(p < 0.05) or **(p < 0.01).

The influence of host-guest interactions on the degree of cell surface functionalization was examined on MDAMB231 × 4 cells. Here for the conditions of the first incubation step were varied as follows; (1) by omitting the use of a CXCR4-binding peptide, (2) by using non-Ad functionalized Ac-TZ14011 (includes a lower affinity tyrosine (Tyr10) guest moiety^47^,^48^) or (3) via the standard procedure by using **Ac-TZ14011-Ad.** Differences in functionalization using **Cy5**_**0.5**_**CD**_**10**_**PIBMA**_**39**_ or **Cy5**_**0.4**_**PIBMA**_**39**_ were analyzed using both semi-quantitative (confocal microscopy) and quantitative (flow cytometry) methods. Under a direct comparison at baseline the non-specific uptake of **Cy5**_**0.5**_**CD**_**10**_**PIBMA**_**39**_ is about one-and-a-half times that of **Cy5**_**0.4**_**PIBMA**_**39**_, which indicates that β-CD can interact with cell-surface residues. Pre-targeting based introduction of guest moieties on the CXCR4 receptors yielded statistically significant (p < 0.01) increases in **Cy5**_**0.5**_**CD**_**10**_**PIBMA**_**39**_ binding (Fig. 3a). The middle column of Fig. 3a, indicates that the Tyr10 residue on the Ac-TZ14011 peptide (Fig. 1b) already induces enhanced binding of the CD-polymer^41^. The introduction of the higher affinity Ad-guest molecule (**Ac-TZ14011-Ad**), further enhances this effect (Fig. 3a, last column).

When the polymer did not include β-CD (host) functionalizations (**Cy5**_**0.4**_**PIBMA**_**39**_), binding was not induced by the presence of **Ac-TZ14011**, or **Ac-TZ14011-Ad** (Fig. 3b). Figure S15 further illustrates that the availability of multiple CD-moieties on the polymer backbone enhances the binding considerably. On average **Cy5**_**0.5**_**CD**_**10**_**PIBMA**_**39**_displayed two-fold higher binding than **Cy5**_**0.4**_**PIBMA**_**39**_. The findings of Fig. 3a,b and supplementary Fig. S15 combined suggest that both guest and host moieties play an instrumental role in the cell functionalization process.

During the (supramolecular) cell surface modification it is expected that one polymer interacts with multiple **Ac-TZ14011-Ad** moieties to establish functionalization. Individual CXCR4 receptors have a diameter of approximately 4 to 5 nm, based on the crystal structure of CXCR4 obtained from the RCSB protein data bank (PDB code 3OE0)^41^. Although the distance between CXCR4 receptors on the membrane is unknown, it is reported that they can cluster in groups^49^,^50^. When assuming a spherical structure, **Cy5**_**0.5**_**CD**_**10**_**PIBMA**_**39**_has a hydrodynamic diameter of 2.8 nm in water but, when unfolded, the polymer length is approximately 24 nm (based on the estimated bond lengths of one subunit, times the number of subunits in the polymer). Hypothetically, this should allow simultaneous interactions with multiple (clustered) **Ac-TZ14011-Ad** functionalized CXCR4 receptors. The longer **Cy3**_**1.5**_**CD**_**72**_**PIBMA**_**389**_ polymer (hydrodynamic diameter ~11.7 nm; unfolded >200 nm) should allow such multivalent interactions even more. To test this theory, the functionalization was also performed using monovalent **Cy5-CD** (**5**) instead of a **CD**_**n**_**PIBMA**_**m**_ polymer, which resulted in a substantial lower degree of functionalization (Supplementary Fig. S14). These findings show that multivalent interactions between β-CD-host molecules and different Ad-guest molecules are indeed required. Furthermore it confirms the assumption that each polymer interacts with at least two or more **Ac-TZ14011-Ad** functionalized CXCR4 receptors.

Since **Cy3**_**1.5**_**CD**_**72**_**PIBMA**_**389**_ differs considerably from **Cy5**_**0.5**_**CD**_**10**_**PIBMA**_**39**_ in length (unfolded; 24 vs. > 200 nm) and in CD number (10 vs. 72), **Cy3**_**1.5**_**CD**_**72**_**PIBMA**_**389**_ can, in theory, bind to more **Ac-TZ14011-Ad** groups than **Cy5**_**0.5**_**CD**_**10**_**PIBMA**_**39**_. This difference was indeed reflected in the affinity of both polymers for the Ad-functionalized cell surfaces. In competition experiments (see supporting information for more detailed description and discussion), **Cy3**_**1.5**_**CD**_**72**_**PIBMA**_**389**_ bound in slightly larger quantities to the cells surface then **Cy5**_**0.5**_**CD**_**10**_**PIBMA**_**39**_ (Supplementary Fig. S17). Competition followed over time by confocal microscopy, revealed that under competitive conditions **Cy3**_**1.5**_**CD**_**72**_**PIBMA**_**389**_ could replace **Cy5**_**0.5**_**CD**_**10**_**PIBMA**_**39**_ cell functionalizations, while the reverse proved to be difficult (Supplementary Figs S18 and S19).

To investigate if the observed replacement is indeed based on host-guest interactions, the same longitudinal competition experiment was repeated with **Cy5**_**0.4**_**PIBMA**_**39**_. This experiment demonstrated that **Cy5**_**0.4**_**PIBMA**_**39**_was not replaced by **Cy3**_**1.5**_**CD**_**72**_**PIBMA**_**389**_, which displayed increased binding to the cell surface over time (see supporting information for more detailed description and discussion; Figs S18 and S19). When the reverse was attempted, **Cy3**_**1.5**_**CD**_**72**_**PIBMA**_**389**_ could also not be replaced by **Cy5**_**0.4**_**PIBMA**_**39**_ while the backbone polymer already displayed binding at an early time-point. These results indicate that the non-specific binding of **Cy5**_**0.4**_**PIBMA**_**39**_ occurs at a different location than where **Cy3**_**1.5**_**CD**_**72**_**PIBMA**_**389**_ binds in a specific manner. These control experiments underline that the observed replacement between **Cy5**_**0.5**_**CD**_**10**_**PIBMA**_**39**_ and **Cy3**_**1.5**_**CD**_**72**_**PIBMA**_**389**_ (Supplementary Figs S18 and S19) represent competition observed in the host-guest interactions between the CD-moieties on the polymers and the **Ac-TZ14011-Ad** ligands.

### Using cells as chemical scaffold for further functionalization

**Figure 4:**
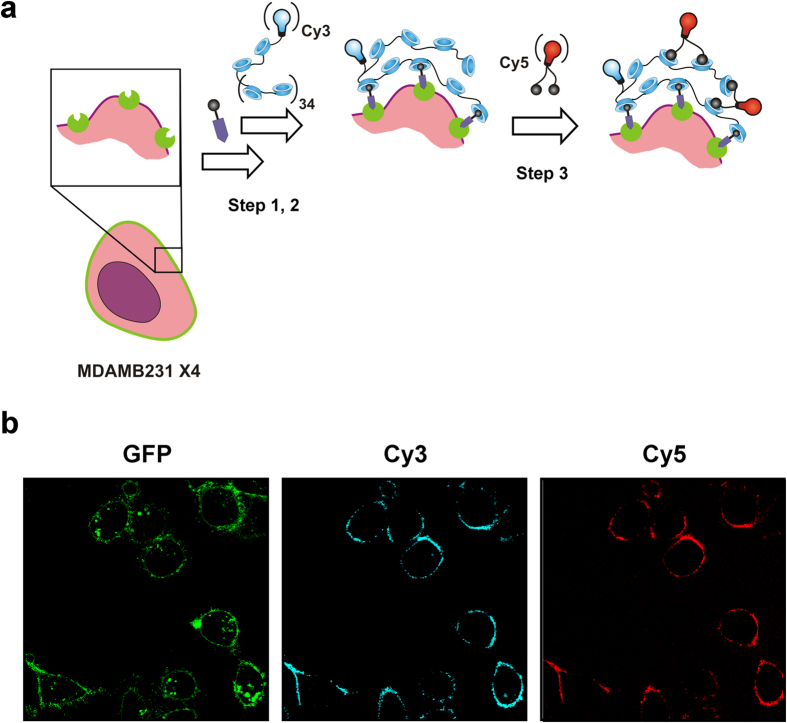
(**a**) Schematic illustration of introducing a third-generation of surface modification, e.g. **Cy5-Ad**_**2**_. The host-guest interaction of CD-Ad is dynamic and after functionalizing the cell surface with CD_n_PIBMA_m_ polymers, e.g. **Cy3**_**1.5**_**CD**_**72**_**PIBMA**_**389**_ (step 1,2), non-bound β-CD groups should be available to host the second fluorescent label (step 3). (**b)** Confocal images visualizing the introduction of **Cy5-Ad**_**2**_ on **Cy3**_**1.5**_**CD**_**72**_**PIBMA**_**389**_ functionalized MDAMB231 × 4 cells. For clarity, both the (overlay) image and the same image at the individual channels are displayed, with GFP in green, Cy3 (**Cy3**_**1.5**_**CD**_**72**_**PIBMA**_**389**_) in blue and Cy5 (**Cy5-Ad**_**2**_) in red.

β-CD based binding of the PIBMA-polymers to the **Ac-TZ14011-Ad** functionalized cell surface is dynamic and occurs with the presence of an excess of β-CD groups on the polymers (Fig. 4a, step 1, 2). Hence, it is expected that non-bound β-CD groups remain available that can be used for consecutive supramolecular functionalization steps. This concept was initially studied using **Cy5-Ad** (**6**) and **Cy5-Ad**_**2**_ (**7**). The monovalent **Cy5-Ad** showed very little staining of cells that were pre-functionalized with **Cy3**_**1.5**_**CD**_**72**_**PIBMA**_**389**_ (Supplementary Fig. S20). In contrast, the bivalent **Cy5-Ad**_**2**_ showed clear staining under identical conditions, providing co-localization of the CXCR4 receptor (GFP), **Cy3**_**1.5**_**CD**_**72**_**PIBMA**_**389**_ (Cy3), and **Cy5-Ad**_**2**_ (Cy5) (Fig. 4b). Use of **Cy5** (**8**) alone did not result in staining (Supplementary Fig. S20). Moreover, experiments where **Cy5-Ad**_**2**_ was added in the absence of **Cy3**_**1.5**_**CD**_**72**_**PIBMA**_**389**_, did not yield non-specific staining which is a clear indication that the polymer is essential for functionalization (Supplementary Fig. S21). When looking at the binding constant of mono- and bivalent Ad with multivalent β-CD hosts in general^51^,^52^,^53^,^54^, a difference of at least a factor 200 is found. The binding constant of bis-adamantane (e.g. **Cy5-Ad**_**2**_) with multivalent β-CD hosts lies between 1·10^7^–1·10^10^ M^−1^ (depending on the host and its environment)^52^,^53^, while the **Cy5-Ad** interaction with **Cy3**_**1.5**_**CD**_**72**_**PIBMA**_**389**_ can be seen as a monovalent interaction of which the binding constant lies around 5·10^4^ M^−1^ 51,54. Again multivalency seems to be a key component for facilitating stable interactions under *in vitro* conditions. The ability to utilize β-CD functionalized cell surfaces to introduce a third-generation of functionalization opens up a scale of functionalization types to tailor a wide range of applications. For example, diagnostic labels for cell-tracking^55^ could be introduced via this route. Alternatively, the introduction of therapeutic agents or a combination of both is possible. Hereby cells are converted into functional scaffolds that can be applied for delivery applications.

**Figure 5:**
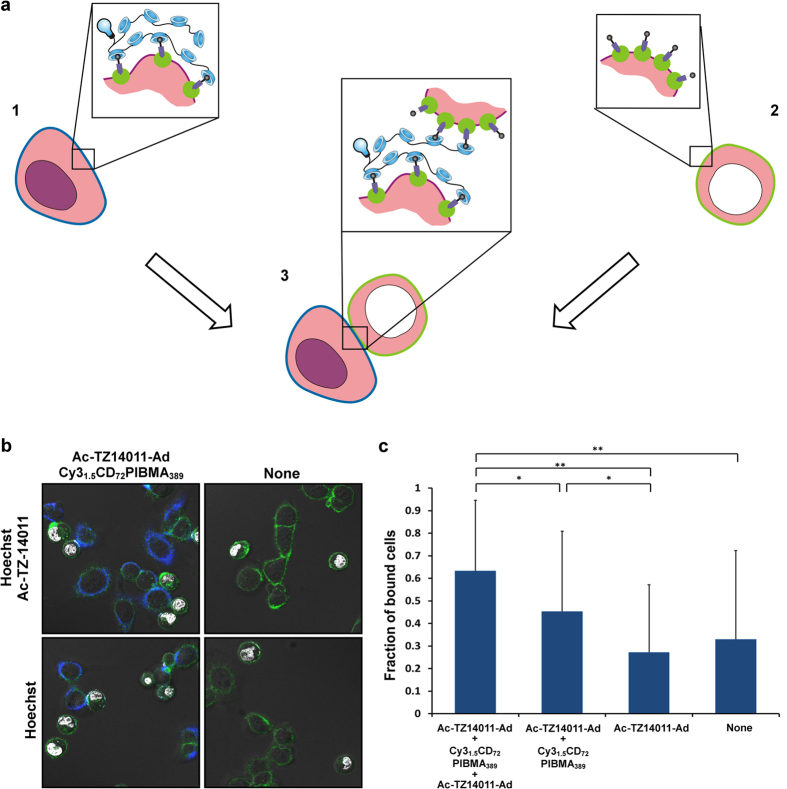
(**a**) Schematic overview of inducing cell-cell interactions (3) between β-CD polymer (**Cy3**_**1.5**_**CD**_**10**_**PIBMA**_**389**_) functionalized cells (1) and Ad (**Ac-TZ14011-Ad**) functionalized cells (2) with Hoechst staining (white) (**b**) Representative confocal images of inducing supramolecular cell-cell interactions between variable functionalized MDAMB231 × 4 cells. With GFP in green, Cy3 in blue and Hoechst in white. (**c**) Average values of the fraction of cell-cell interactions in each test condition. Significance of differences is marked with *(p < 0.05) or **(p < 0.01).

Given the fact that the CD_n_PIBMA_m_ polymers interact with **Ac-TZ14011-Ad** functionalization on the cell surface and that the secondary polymer surface functionalization enables a third-generation of surface modifications, we reasoned that it would be of interest to use such technology to drive the interactions between MDAMB231 × 4 cells that are either functionalized with CD_n_PIBMA_m_ polymers or **Ac-TZ14011-Ad** (Fig. 5a).

To study the induction of cell-cell interactions, **Ac-TZ14011-Ad** + **Cy3**_**1.5**_**CD**_**72**_**PIBMA**_**389**_ functionalized adhered MDAMB231 × 4 cells were incubated with a solution containing **Ac-TZ14011-Ad** functionalized MDAMB231 × 4 cells in suspension (see Fig. 5a for a schematic representation). In the latter the nucleus was stained with Hoechst in order to enable discrimination between the two. After 15–30 min of incubation, cell-cell interactions were quantified using confocal microscopy (Fig. 5b). Analysis of the obtained images revealed that on average 61% of the Hoechst stained suspended cells within the field of view interacted with non-Hoechst stained adherent cells. Control experiments where the adherent cells were not functionalized using **Cy3**_**1.5**_**CD**_**72**_**PIBMA**_**389**_and/or in which the cells in suspension were not functionalized with **Ac-TZ14011-Ad** resulted in significantly (p < 0.01 and p < 0.05 respectively) lower percentages of cell-cell interactions, as is depicted in Fig. 5. This made us conclude that the introduced cell-surface modifications and underlying supramolecular chemistry opens the perspective to drive cell-cell interactions.

Synthetic control on cell-cell enhancing interactions could be beneficial for cell-based therapies^7^,^8^,^9^. For example, a challenge in (heart) stem-cell transplantation is to make the cells reside at the site of interest long enough to deliver a therapeutic effect^10^. In the current clinical set-up, for example, cardiac stem cells are quickly cleared from location after intramyocardial injection^56^. If the interaction of a transplanted cell with its surrounding could be enhanced, e.g. by providing a “temporary glue-like” adhesion of the cells at the injection site, the local retention could be improved. By allowing the cells time to engraft to the host tissue using natural transmembrane receptor interactions, the cellular retention and thus the therapeutic efficacy is likely to be enhanced. Alternatively, the same mechanism could be applied to temporarily adhere cells that excrete therapeutic substances such as enzymes^57^. To demonstrate that the technology described is not limited to cancer cells we successfully applied this technology on CXCR4 expressing human cardiac stem cells (Supplementary Fig. S22), which are currently used in stem cell-therapy. After having established all the chemical requirements for the supramolecular cell-surface modification, studies regarding the biological efficacy of functionalized stem cells will be initiated.

The cell-surface modification approach as described in this manuscript, obtains its cell-type specificity from the specific targeting of membrane receptors, in this case being CXCR4 (Fig. 1, 1). While polymer modification of the cell surface is a generic step (Fig. 1, 2), the introduction of functionalities for e.g. adhesion can again be tailored if required. This provides a large degree of (synthetic) freedom and possibilities. With the many membrane-receptor targeting vectors available on the market these days^38^,^40^,^43^,^58^,^59^, and the huge variety of functionalities that could be of value, the proposed approach can be made compatible with a whole scale of cells and cell-therapy applications. The most critical part herein seems to be a high local density of one, or a combination of, membrane-receptors so that multivalent interactions with the host polymer are possible.

Based on the possibility of introducing specificity via the membrane-receptors (Fig. 2, 3) it may be postulated that the supramolecular approach provides a good alternative for current cell surface functionalization methods, such as the layer by layer (LBL) a-specific cell coating by polyelectrolyte polymers^18^ or the insertion of lipophilic anchors^20^. While the LBL technique has proven its applicability as cell coating on different sturdy cell membranes, e.g. bacteria and pancreatic islets^60^, the supramolecular functionalization is more subtle and allows cell surface modification in more sensitive cell types without harming the cell viability (Supplementary Fig. S13).

## Methods

### General

For information on the materials used and more in depth experimental descriptions (including compound synthesis and analysis, determination of the receptor affinity of Ac-TZ14011-Ad, cell culture and confocal microscopy), see the Supporting information (SI).

### Polymer synthesis

Poly(isobutylene-alt-maleic anhydride) (PIBMA_39,_ M_w_ 6,000) or PIBMA_389_ (M_w_ 60,000) were dissolved in dry DMSO together with DIPEA and the appropriate Cy5- or Cy3-dye. The reaction was left to stir for at least 7 h. Then 6-monodeoxy-6-monoamine-β-cyclodextrin (β-CD) was added and the mixture was stirred at 80 °C for another 12 h. After cooling to RT, the polymer was dialyzed against H_2_O for 1 day, then against 100 mM phosphate buffer pH 9.0 for another day, and finally against H_2_O for 5 days. The dialysis medium was refreshed every day. The remaining solution was then lyophilized to obtain the product. The number of CD groups per polymer, was estimated via ^1^H NMR analysis and the number of dyes per polymer was estimated via UV/Vis absorbance (see Supporting information). Prior to use, a solution of 1 mg/mL in H_2_O each polymer was prepared and stored at 4 °C.

#### Cy5_0.4_ PIBMA_39_ (compound **2**)

PIBMA_39_ (9.1 mg, 1.5 μmol) and Cy5-Sulfonate-Amine (**10**) (1 mg, 1.8 μmol) were dissolved in 0.6 mL dry DMSO, DIPEA (13 μL, 74 μmol) was added and the mixture was stirred at RT overnight. Subsequently, the reaction mixture was directly dialyzed and after lyophilization, the product was obtained as a blue powder (2 mg, 0.3 μmol).

Average number of Cy5 dye/polymer according UV/vis absorbance: 0.4

Estimated molecular weight: 7.8 kDa (Table S1).

#### Cy5_0.5_CD_10_PIBMA_39_ (compound **3**)

PIBMA_39_ (30 mg, 5 μmol) and Cy5-(SO_3_)Sulfonate-(SO_3_)Amine (**9**) (5.0 mg, 5.6 μmol) were dissolved in 3 mL dry DMSO and DIPEA (50 μL, 250 μmol) was added. The reaction was stirred at 80 °C for 7 h, then 6-monodeoxy-6-monoamino-β-cyclodextrin (95 mg, 80 μmol) was added and the solution was stirred for another 72 h at 80 °C. After dialysis and lyophilization, the product was obtained as a blue powder (87 mg, 5 μmol).

Average number of CD groups/polymer according ^1^H NMR: 10

Average number of Cy5 dye/polymer according UV/vis absorbance: 0.5

Estimated molecular weight: 18.8 kDa (Table S1).

#### Cy3_1.5_CD_72_PIBMA_389_ (compound **4**)

PIBMA_389_ (10 mg, 0.17 μmol) and DIPEA (15 μL, 85 μmol) were dissolved in 1.7 mL dry DMSO and a solution of Cy3-Amine-COOH (**11**) in dry DMSO (0.25 mM, 800 μL, 0.2 μmol) was added. The reaction was stirred overnight at RT, then, 6-monodeoxy-6-monoamino-β-cyclodextrin (31.6 mg, 27 μmol) was added and the solution was stirred for another night at 80 °C. After dialysis and lyophilization the product was obtained as a bright pink powder (22.3 mg, 0.16 μmol).

Average number of CD groups/polymer according ^1^H NMR: 72

Average number of Cy3 dye/polymer according UV/vis absorbance: 1.5

Estimated molecular weight: 155 kDa (Table S2).

### Cell experiments

#### Functionalization of cells

MDAMB231 × 4, were seeded onto culture dishes (80,000 per dish) and brought to 0 °C, followed by incubation with **Ac-TZ14011-Ad** (10 μM) in 1 mL DMEM for 1 h at 0 °C. Subsequently, either **Cy5**_**0.5**_**CD**_**10**_**PIBMA**_**39**_ or **Cy3**_**1.5**_**CD**_**72**_**PIBMA**_**389**_ was added (10 μM final β-CD concentration). After 1 h of incubation at 0 °C, cells were washed twice with PBS and confocal images were taken.

#### Functionalization of cells in a mixed cell-culture

A mixed-cell set-up was used to determine the specificity of the cell functionalization. A mixture of 40,000 cells of each strain of MDAMB231 × 4 and MDAMB231 cells were seeded. The next day, the cells were brought to 0 °C and subsequently they were functionalized with either **Cy5**_**0.5**_**CD**_**10**_**PIBMA**_**39**_ or **Cy3**_**1.5**_**CD**_**72**_**PIBMA**_**389**_ (see functionalization of the cells). During confocal analysis discrimination between the fluorescence of the outer membrane of the two cell lines was based on the GFP signal, only present in the MDAMB231 × 4 strain. To determine the difference in polymer binding to **Ac-TZ14011-Ad** functionalized MDAMB231 × 4 and MDAMB231 cells, the experiment was performed twice and for each cell type the average gray value/m^2^ of 25 cells in total were measured (see SI for further details).

#### Ac-TZ14011-Ad induced cell functionalization analyzed by confocal microscopy

The receptor-mediated functionalization of cells was examined by confocal microscopy and flow cytometry (see also **‘Ac-TZ14011-Ad** induced cell functionalization analyzed by flow cytometry’). For Confocal microscopy MDAMB231 × 4 cells (80,000 per well) were incubated with either Ac-TZ14011 (10 μM), **Ac-TZ14011-Ad** (10 μM), or none, for 1 h at 0 °C in 1 mL DMEM. Subsequently, either **Cy5**_**0.4**_**PIBMA**_**39**_ or **Cy5**_**0.5**_**CD**_**10**_**PIBMA**_**39**_ was added (10 μM β-CD; 1 μM polymer final concentration) and another hour at 0 °C of incubation followed. Thereafter, the cells were washed twice with PBS and confocal images were acquired. All experiments were performed in 6-fold and for each condition per experiment at least 10 cells were included in the study. The Cy5 signal present on the cell in each sample was quantified to analyze differences between the amount of binding of the polymers to the cells when either, no peptide, Ac-TZ14011, or **Ac-TZ14011-Ad** was present. For normalization all results were divided by the average fluorescence value obtained when just the polymer was added. The significance of the obtained differences was determined by student T-test (two tailed, unpaired).

#### Cy5-Ad and Cy5-Ad_2_ functionalization of polymer modified cell surfaces

A third functionalization on the β-CD-polymer functionalized cells was introduced, by first functionalizing adherent MDAMB231 × 4 cells with **Cy3**_**1.5**_**CD**_**72**_**PIBMA**_**389**_. Subsequently, the cells were washed once with DMEM, followed by incubation with **Cy5-Ad**_**n**_ (n = 1 or 2, 5 μM) in 1 mL DMEM for 1 h at 0 °C. Before confocal images were taken, the cells were washed twice with PBS. As a control experiment, the cells were incubated with **Cy5-Ad**_**2**_ (5 μM final concentration) while the polymer was omitted in the first incubation step.

#### Cell-cell interactions

To study cell-cell interactions, variable combinations of functionalized MDAMB231 × 4 cells were evaluated. MDAMB231 × 4 cells (300,000 per tube) in suspension were incubated with Hoechst 33342 (1 μg/mL) for 30 minutes in 1 mL DMEM at 0 °C. Subsequently, they were washed once with PBS (centrifuged 3 min, 3000 × *g*, 4 °C), cooled on ice and either incubated with **Ac-TZ14011-Ad** (11 μM) or none, in DMEM (500 μL) for 1 h at 0 °C. After washing twice with PBS (centrifuged 3 min, 3000 × *g*, 4 °C), cells were resuspended in 300 μL PBS and added to a separate batch of adherent MDAMB231 × 4 target cells (80,000 cells per dish). The latter were either functionalized with **Cy3**_**1.5**_**CD**_**72**_**PIBMA**_**389**_ or none, and subsequently washed with PBS. The variable cell mixtures, see Table S1, were allowed to incubate in 1 mL PBS for 15 to 30 min at RT. Prior to imaging, the excess of unbound cells in suspension were gently washed away with PBS (2 × 1 mL, RT).

The samples were examined under confocal microscopy in a culture dish, of each sample approximately 10 images were acquired at randomly chosen locations. All experiments were performed in 5-fold, resulting in the analysis of 223 ± 30 cells per cell combination. For each image obtained, the ratio between Hoechst stained cells that had an interaction with a target cell and the total number of Hoechst-stained cells in the image, was calculated. Obtained ratio’s for each cell combination (Table 1) were averaged and statistical significance of differences between each cell combination determined using student T-test (two tailed, unpaired).

#### Ac-TZ14011-Ad induced cell functionalization analyzed by flow cytometry

Besides examining the receptor-mediated functionalization of cells by confocal microscopy (see **‘Ac-TZ14011-Ad** induced cell functionalization analyzed by confocal microscopy’) the functionalization was also examined by flow cytometry to quantify the rate of functionalization. For this purpose, MDAMB231 × 4 cells were trypsinized and divided into aliquots (300,000 cells per tube), centrifuged for three minutes (3000 × *g*, 4 °C), and supernatant was decanted. The cells were incubated with 50 μL PBS containing either Ac-TZ14011 (10 μM), **Ac-TZ14011-Ad** (10 μM), or none for 1 h at 0 °C. Subsequently, 50 μL of either **Cy5**_**0.4**_**PIBMA**_**39**_ or **Cy5**_**0.5**_**CD**_**10**_**PIBMA**_**39**_ in PBS was added (10 μM β-CD; 1 μM polymer final concentration) and another hour at 0 °C of incubation followed. The cells were washed twice with PBS (centrifuged 3 min, 3000 × g, 4 °C), resuspended in 300 μL PBS and the intensity of Cy5 fluorescence related to the cells was measured by flow cytometry (see ESI for further details). All experiments were performed in 8-fold. For data normalization, all results were divided by the average fluorescence value obtained when only the polymer was added. The significance of the obtained differences was determined by student T-test (two tailed, unpaired).

## Conclusions

In this study we have shown that cell specific sequential surface functionalization could be accomplished by pre-targeting cells with an Ad-containing targeting vector such as **Ac-TZ14011-Ad**. This pre-targeting approach provides the possibility of tailoring this first step towards other cell types. Subsequently, host-guest interactions between fluorescent labeled **CD_n_PIBMA_m_** host polymers and the introduced Ad-guest functionality not only enable surface coating, but also formed a basis for further hierarchical functionalization.

The described host-guest approach adds the possibility to introduce specific functionalizations on the **CD_n_PIBMA_m_** modified cell surface. There are always non-bound β-CD groups available for further functionalization, since the polymers contain an access of β-CD groups. The specific functionalizations can be in the form of diagnostic- and/or therapeutic-labels, so that the cells become vehicles for imaging and/or drug-delivery applications. In addition, we have shown that theses supramolecular functionalizations provide a basis to drive cell-cell interactions that could prove to be of future benefit for cell based therapies.

## Additional Information

**How to cite this article**: Rood, M. T. M. *et al*. Obtaining control of cell surface functionalizations via Pre-targeting and Supramolecular host guest interactions. *Sci. Rep.* **7**, 39908; doi: 10.1038/srep39908 (2017).

**Publisher's note:** Springer Nature remains neutral with regard to jurisdictional claims in published maps and institutional affiliations.

## Supplementary Material

Supplementary Information

## Figures and Tables

**Table 1:** Tested cell mixtures of adhered and suspended MDAMB231 X4 cells and their functionalization.

Cell mixture	Adherent MDAMB231 X4	In suspension MDAMB231 X4 + Hoechst labelled
1	**Ac-TZ14011-Ad** + **Cy3**_**1.5**_**CD**_**72**_**PIBMA**_**389**_	**Ac-TZ14011-Ad**
2	**Ac-TZ14011-Ad** + **Cy3**_**1.5**_**CD**_**72**_**PIBMA**_**389**_	None
3	None	**Ac-TZ14011-Ad**
4	None	None
