# Supplementary material for: Obtaining control of cell surface functionalizations via Pre-targeting and Supramolecular host guest interactions
Source: Sci Rep. 2017 Jan 6;7:39908. doi: 10.1038/srep39908 (PMC5216351; doi:10.1038/srep39908)
Supplement: Supplementary Information [file srep39908-s1.pdf]

Supporting information

Obtaining control of cell surface functionalizations via Pre-targeting and Supramolecular host guest interactions

Mark T.M. Rood,† Silvia J. Spa,† Mick M. Welling,† Jan Bart ten Hove,‡ Danny M. van Willigen,† Tessa Buckle,† Aldrik H. Velders,‡ Fijs W.B. van Leeuwen*,†

†Interventional Molecular Imaging Laboratory, Department of Radiology, Leiden University Medical Center, Albinusdreef 2, PO BOX 9600, 2300 RC, Leiden, The Netherlands

‡Laboratory of BioNanoTechnology, Axis, Building 118, Bornse weilanden 9, 6708 WG Wageningen, The Netherlands

Experimental Section

Materials

All chemicals were obtained from commercial sources and used without further purification. ISOBAM-04 was kindly supplied by Kuraray Europe GmbH free of charge. NMR spectra were recorded using a Bruker DPX 300 spectrometer (300 MHz ^{1}H NMR) or a Bruker AVANCE III 500 MHz with a TXI gradient probe and are referenced to residual solvent signal or TMS. HPLC was performed on a Waters system by using a 1525EF pump and a 2489 UV detector. For the MTT assay the Perkin Elmer plate reader 1420 Multilabel Counter was applied. For preparative HPLC a Dr. Maisch GmbH, Reprosil-Pur 120 C18-AQ 10 µm (250×20 mm) column was used and a gradient of 0.1 % TFA in H_2O/CH_3CN (95:5) to 0.1 % TFA in H_2O/CH_3CN (5:95) in 40 min was employed. For analytical HPLC a Dr. Maisch GmbH, Reprosil-Pur C18-AQ 5 µm (250×4.6 mm)

column was used and a gradient of 0.1 % TFA in H_2O/CH_3CN (95:5) to 0.1 % TFA in H_2O/CH_3CN (5:95) in 40 min was employed. MALDI-TOF measurements were performed on a Bruker Microflex. For dialysis Sigma Pur-A-Lyzer^{TM} Mega 3500 tubes were used.

Synthesis of the cyanine-dye building blocks

Indole

The indole-based building blocks; Indole-Sulfonate, Indole-COOH, sulfoindole-Sulfonate and sulfoindole-COOH were synthesized according a previously reported procedure,[1,2] while the indole-based building blocks; indole-Phth , Indole-AmineBoc, and sulfoindole-AmineBoc were synthesized according an adjusted synthesis method based on published procedure.[2,3] The crude product of the Indole building blocks could be directly used in the next reaction step, except for sulfoindole-AmineBoc, which was purified first.

Indole-Phth

A mixture of 2,3,3-trimethylindolenine (504 µL, 3.1 mmol) and N-(3-Bromopropyl)phthalimide (843 mg, 3.1 mmol) in 5 mL MeCN was stirred for 4 h at 100 ^oC, followed by 72 h at 60 ^oC. The resulting red precipitate was collected, dissolved in acetone and precipitated in Et_2O. The suspension was filtrated and the residue was washed with Et_2O yielding the crude product as an orange solid (1.2 g)

Indole-AmineBoc

A solution of 2,3,3-trimethylindolenine (3.7 ml, 22.8 mmol) and tert-butyl-(3-bromopropyl)carbamate (5.4 g, 22.8 mmol) in 25 ml dry MeCN was stirred for 72 h at 60 ^oC. The mixture was concentrated under vacuum, re-dissolved in a small amount of MeOH and precipitated in Et_2O while stirring. The precipitate was filtered off and washed with Et_2O until the filtrate was colorless, yielding the product as a pink solid (3.5 g).

Sulfoindole-AmineBoc

A solution of 2,3,3-trimethyl-3H-indole-5-sulfonate potassium salt (1.1 g, 4 mmol) and 3-propylamine·HBr (0.87 g, 4 mmol) in 10 mL 1,2-dichlorobenzene was stirred for 30 min at 110 ^{o}C, followed by 10 min at 150 ^{o}C. The resulting purple precipitate was collected and dispersed in 15 mL MeOH. Di-tert-butyldicarbonate (1.7 g, 8 mmol) and DIPEA (1.4 mL, 8 mmol) were added and the reaction mixture was refluxed for 30 minutes. The mixture was concentrated in vacuo and purified by column chromatography (MeOH:CH_2Cl_2 1:3), yielding the product as a pink solid (81 mg).

Synthesis of the cyanine-dyes

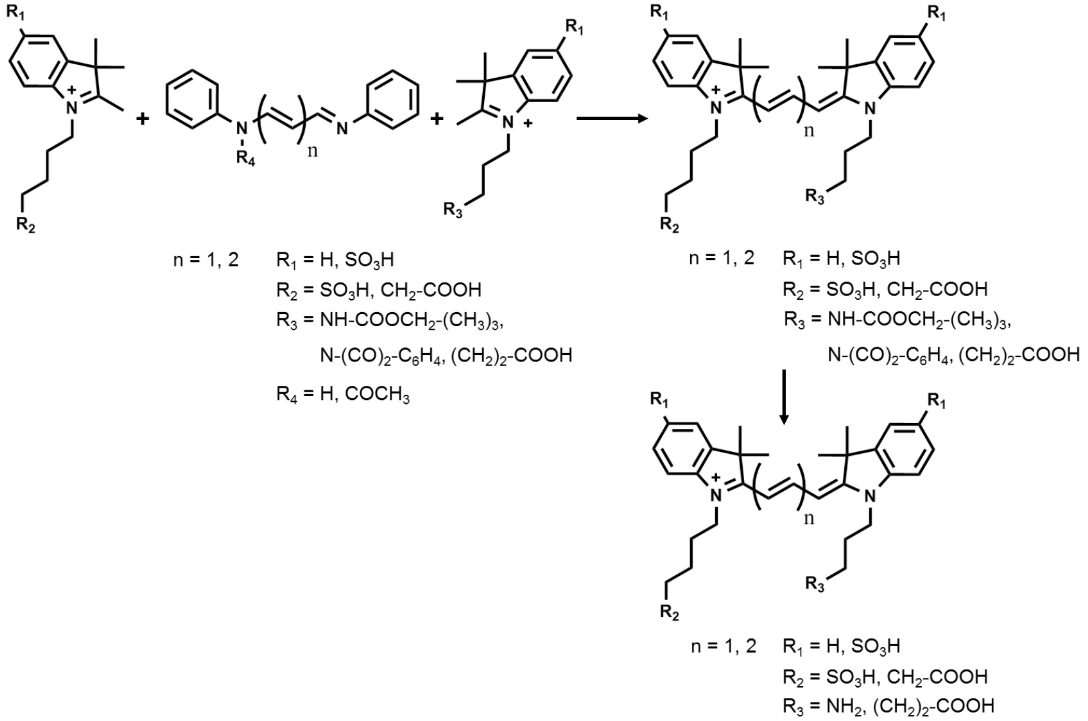

Figure S1: Synthesis of Cy5-(SO_3)Sulfonate-(SO_3)COOH (compound **8**) (n = 1, R_1 = SO_3H, R_2 = SO_3H, R_3 = $(CH_2)_2$-COOH), Cy5-(SO_3)Sulfonate-(SO_3)Amine (compound **9**), Cy5-Sulfonate-Amine (compound **10**) (n = 1, R_1 = H, R_2 = SO_3H, R_3 = NH_2), and Cy3-Amine-COOH (compound **11**) (n = 0, R_1 = H, R_2 = CH_2-COOH, R_3 = NH_2), (n = 1, R_1 = SO_3H, R_2 = SO_3H, R_3 = NH_2).

*Cy5-(SO_3)Sulfonate-(SO_3)COOH (compound **8**)*

Cy5-(SO_3)Sulfonate-(SO_3)COOH was synthesized according a previously reported method.[2,4]
MS (MALDI-TOF): $[C_{35}H_{45}N_2O_{11}S_3]^+$ calcd 765.2, found 765.7. ^{1}H NMR spectrum as previously described[2,4]

*Cy5-(SO_3)Sulfonate-(SO_3)Amine (compound **9**)*

Sulfoindole-Sulfonate (41 mg, 0.1 mmol) and 3-anilinoacraldehyde anil hydrochloride (28 mg, 0.1 mmol) were dissolved in 4 mL HOAc:AC_2O (1:1). After 30 minutes at 110 °C, the compound was precipitated in 50 mL diethyl ether. The obtained solid was dissolved in a mixture of 8 mL Ac_2O:Pyridine (1:1) and sulfoindole-AmineBoc (40 mg, 0.1 mmol) was added. After stirring at RT overnight, the product was concentrated under vacuo and purified by preparative HPLC. The product containing fraction was collected and lyophilized to give 5.0 mg (6.2 µmol) of Cy5-(SO_3)Sulfonate-(SO_3)AmineBoc. Subsequently, Cy5-(SO_3)Sulfonate-(SO_3)AmineBoc was deprotected by stirring in TFA:MeCN:CH_2Cl_2 2:2:1 overnight at RT. After evaporation of the solvents, Cy5-(SO_3)Sulfonate-(SO_3)Amine was obtained as a dark blue powder (4.4 mg, 6.2 µmol, 6% yield)
MS (MALDI-TOF): $[C_{32}H_{42}N_3O_9S_3]^+$ calcd 708.1, found 708.2. ^{1}H NMR (300 MHz, D_2O): 8.00 (m, 2H, CH), 7.83-7.74 (dd, 4H, Ar-H), 7.34 (d, 1H, Ar-H), 7.20 (d, 1H, Ar-H), 6.54 (t, 1H, CH), 6.34 (d, 1H, CH), 6.16 (d, 1H, CH), 4.09 (m, 4H, N-CH_2), 3.10 (t, 2H, CH_2-NH_2), 2.96 (t, 2H, CH_2-SO_3), 2.15-1.91 (m, 6H, 3CH_2), 1.61 (d, 12H, C-($\underline{CH_3}$)$_2$) ppm.

*Cy5-Sulfonate-Amine (compound **10**)*

Indole-Sulfonate (100 mg, 0.34 mmol) and 3-anilinoacraldehyde anil hydrochloride (88 mm, 0.34 mmol) were dissolved in 10 mL HOAc:AC_2O (1:1). After 60 min stirring at 110 ^{o}C the mixture was cooled down and a solution of Indole-AmineBoc (161 mg, 0.51 mmol) in 10 mL pyridine was added. After stirring for 2 h at 140 ^{o}C, the blue solution was concentrated under vacuo and purified by column chromatography (MeOH:CH_2Cl_2 1:10 to 1:1 gradient) and preparative HPLC. Product containing fractions were collected and lyophilized to yield a black solid. Subsequently, the obtained solid was dissolved in 30 mL DCM:TFA (1:1) and a few drops of H_2O were added. After 3 h stirring, the reaction mixture was concentrated under vacuo,

redissolved in H_2O and lyophilized to yield the product as a blue powder (11.6 mg, 21 µmol, 4.2% yield)

MS (MALDI-TOF): $[C_{32}H_{42}N_3O_3S]^+$ calcd 548.7, found 548.5. ^{1}H NMR (300 MHz, MeOD): 8.28 (m, 2H, CH), 7.53-7.23 (qt, 8H, Ar-H), 6.69 (t, 1H, CH), 6.47 (d, 1H, CH), 6.26 (d, 1H, CH), 4.18 (m, 4H, N-CH_2), 3.12 (t, 1H, CH_2-NH_2), 2.93 (t, 2H, CH_2-SO_3), 2.16-1.99 (m, 6H, 3CH_2), 1.73 (s, 12H, C-($\underline{CH_3}$)$_2$) ppm.

*Cy3-Amine-COOH (compound **11**)*

Synthesis of Cy3-Amine-COOH was adapted from previously described asymmetric cyanine synthesis.[5] N,N′-Diphenylformamidine (102 mg, 0.52 mmol) was dissolved in cold DCM (50 mL). DIPEA (181 µL, 1.04 mmol) and acetic anhydride (59 µL, 0.63 mmol) were added and the mixture was stirred for 2 h at room temperature. The mixture was concentrated under vacuum yielding a colorless oil. The oil was re-dissolved in 20 mL EtOH together with indole-COOH (127 mg, 0.46 mmol), indole-Phth (197 mg, 0.46 mmol) and pyridine (169 µL, 2.10 mmol). The solution was refluxed for 3 h and stirred overnight at 60 °C. Acetic anhydride (60 µL) was added, and the reaction mixture turned pink. After refluxing for 4 h, the crude product was concentrated in vacuo and purified by column chromatography (eluens: MeOH). The product fractions were combined, concentrated and lyophilized to give a pink solid of impure compound Cy3-Phth-COOH. A portion of Cy3-Phth-COOH (100 mg, 0.16 mmol) was further purified by preparative HPLC. After lyophilization of the product fractions, Cy3-Phth-COOH was deprotected by adding 2 mL of CH_3NH_2 (33% in EtOH). The solution was stirred for 5 h, after which the reaction was concentrated in vacuo to give Cy3-Amine-COOH as a pink solid. Subsequently the compound was purified by preparative HPLC. Fraction containing product was collected and lyophilized and gave the pure product as a pink solid (4 mg, 7.99 µmol, 5% yield).

MS (MALDI-TOF): $[C_{32}H_{42}N_3O_2]^+$ calcd 500.3, found 499.3. ^{1}H NMR (500 MHz, DMSO-d6): 8.36 (t, 1H, CH), 7.66 (d, 2H, Ar-H), 7.51 (d, 2H, Ar-H), 7.46 (t, 2H, Ar-H), 7.32 (q, Ar-H), 6.55 (d, CH), 6.46 (d, 1H, CH), 4.21 (m, 2H, N-CH_2), 4.12 (m, 2H, N-CH_2), 2.94 (m, 2H, CH_2-NH_2), 2.22 (t, 2H, CH_2-SO_3), 2.03 (m, 2H, CH_2), 1.74 (m, 2H, CH_2), 1.71 (d, 12H, C-($\underline{CH_3}$)$_2$), 156 (m, 2H, CH_2), 1.43 (m, 2H, CH_2) ppm.

Polymer synthesis

*$Cy5_{0.4}$ $PIBMA_{39}$ (compound **2**), $Cy5_{0.5}CD_{10}PIBMA_{39}$ (compound **3**), and $Cy3_{1.5}CD_{72}PIBMA_{389}$ (compound **4**)*

For detailed description of the polymers synthesis, see main manuscript

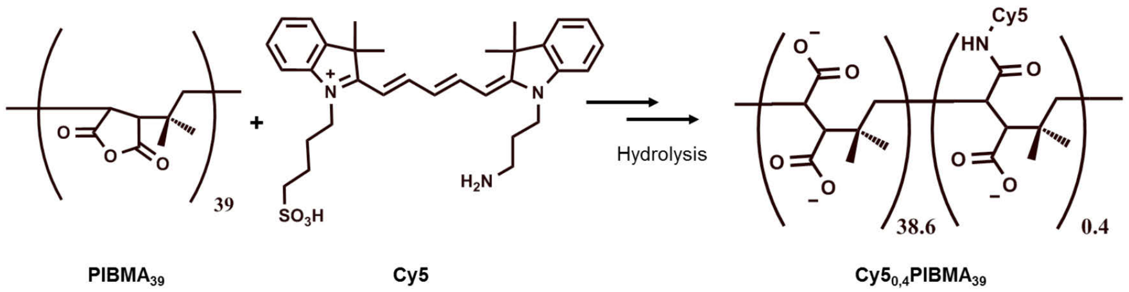

Figure S2: Conjugation of $PIBMA_{39}$ with Cy5-Sulfonate-Amine (**10**).

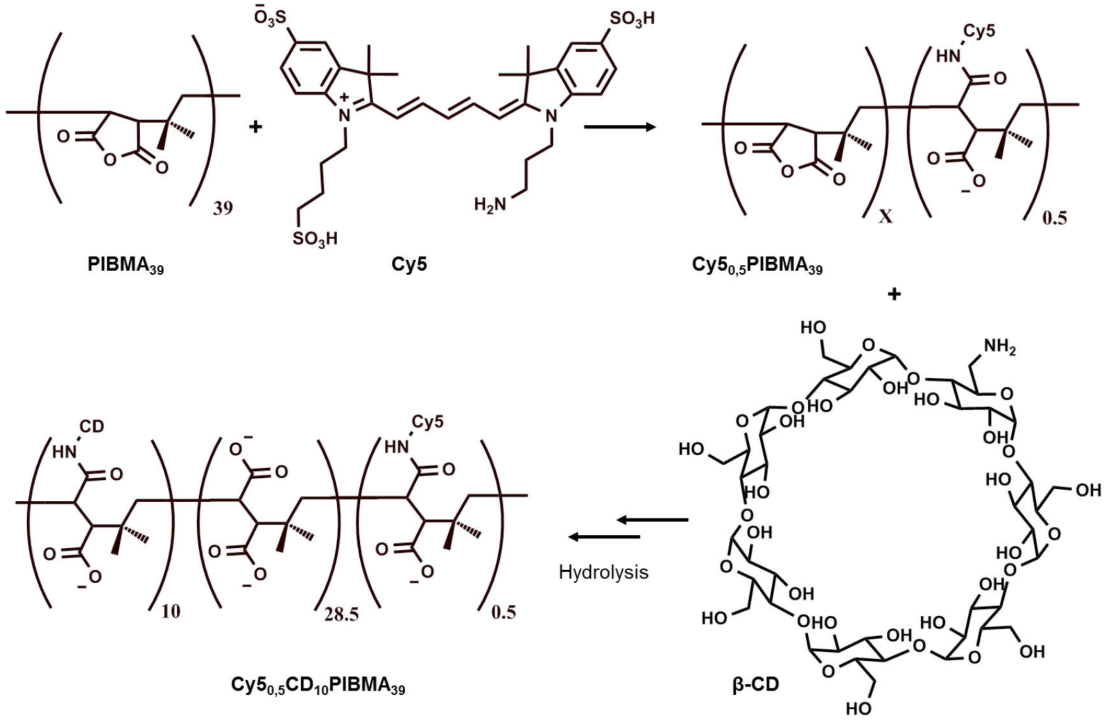

Figure S3: Conjugation of $PIBMA_{39}$ with Cy3-Amine-COOH (**9**) and subsequently with β-CD.

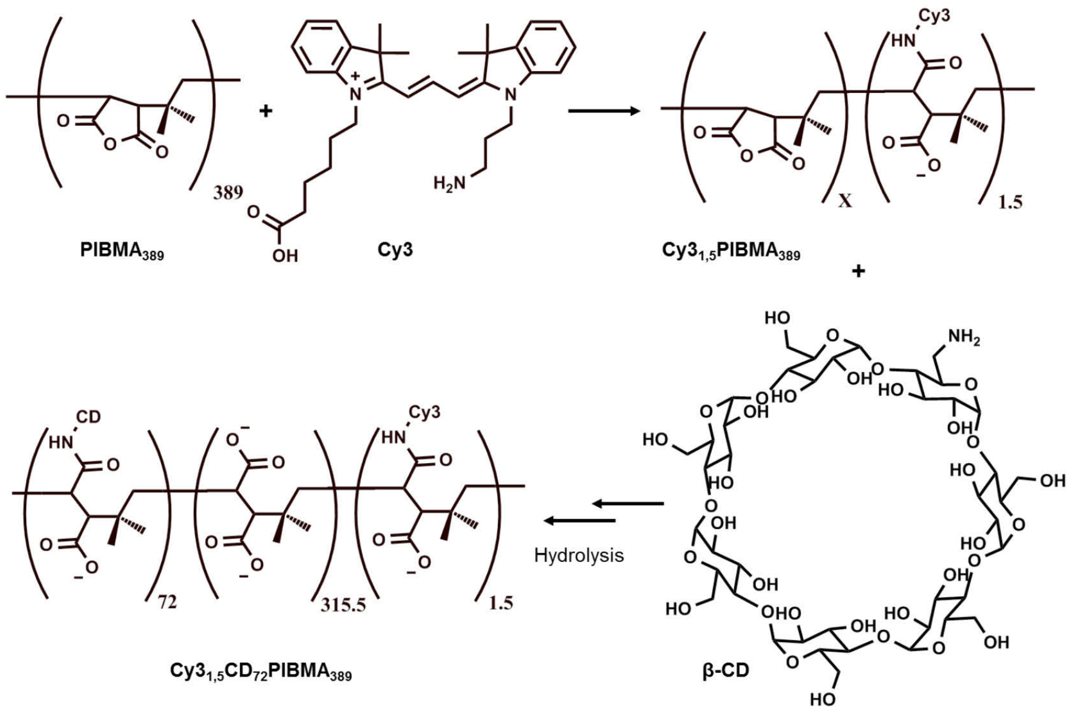

Figure S4: Conjugation of $PIBMA_{389}$ with Cy3-(Sulfonate-(SO_3)Amine (**11**) and subsequently with β-CD.

Synthesis of Ad-functionalized compounds

*Ac-TZ14011-Ad (compound **1**)*

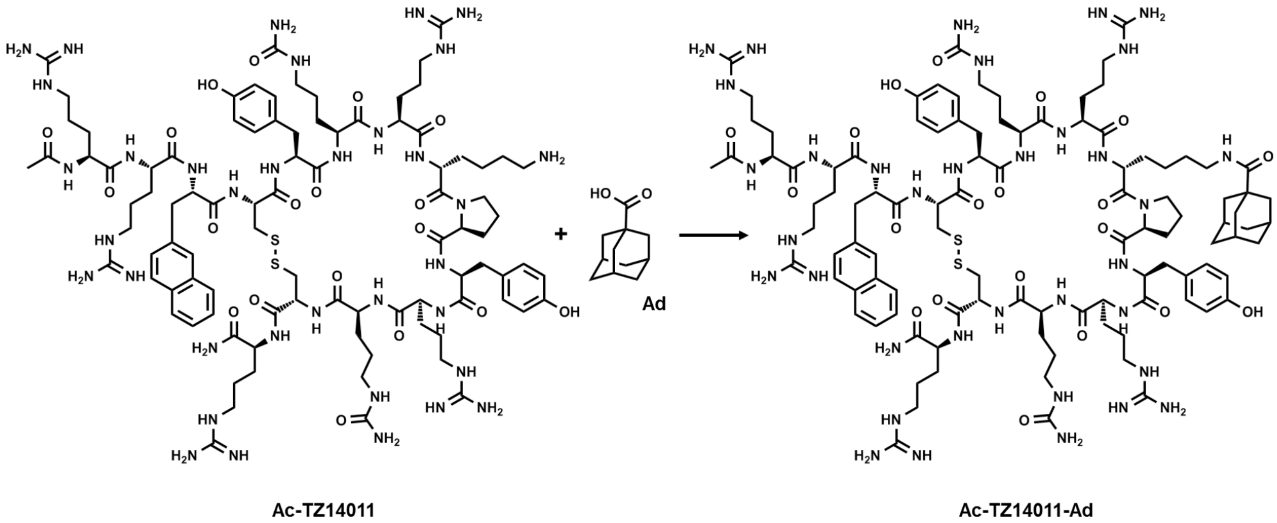

Figure S5: Conjugation of Ac-TZ14011 with 1-adamantanecarboxilic acid

PyBOP (2.8 mg, 5.4 µmol), 1-adamantanecarboxylic acid (1.1 mg, 6 µmol) and DIPEA (5.1 µl, 30 µmol) were dissolved in 1 mL dry DMF and stirred for 5 minutes at RT. This was added to a solution of Ac-TZ14011 (9.8 mg, 3.5 µmol), synthesized as previously described,[6] in 1 mL dry DMF. The reaction mixture was stirred for 48 h at RT. Subsequently, 2.5 mL of 0.1% TFA (H_2O) was added to the reaction mixture to purify the reaction mixture directly by preparative HPLC. The product fraction was lyophilized to give the product as a white powder (8 mg, 2.7 µmol, 77% yield).

MS (MALDI-TOF): $[C_{103}H_{158}N_{35}O_{20}S_2]^+$ calcd 2270.7, found 2271.3. The analytical HPLC chromatogram is shown in Supplementary Fig. S9.

*Cy5-Ad (compound **6**)*

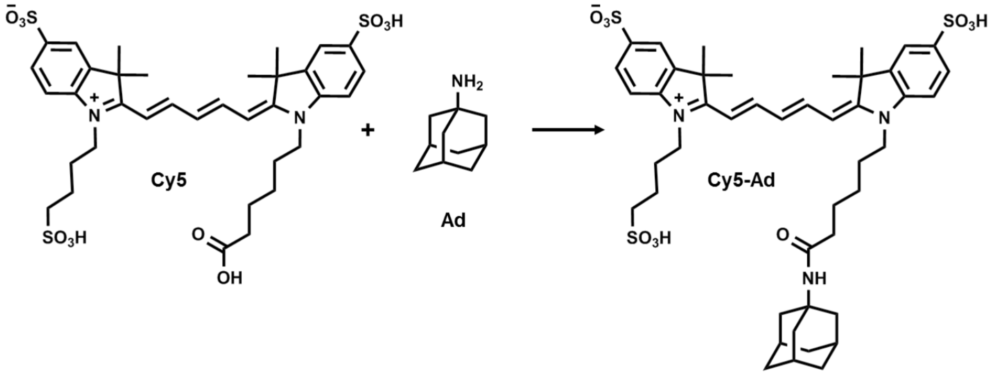

Figure S6: Conjugation of Cy5-(SO_3)Sulfonate-(SO_3)COOH (Cy5, **8**) with aminoadamantane (Ad).

1- Aminoadamantane hydrochloride (7.5 mg, 40 µmol), Cy5-(SO_3)Sulfonate-(SO_3)COOH (**8**) (8.2 mg, 10 µmol) and PyBOP (31 mg, 60 µmol) were dissolved in 2 mL dry DMF. DIPEA (50 µL, 300 µmol) was added and the reaction was stirred overnight, in the dark, at RT. Solvents were evaporated in vacuo and the product was purified by preparative HPLC. The product containing fraction was lyophilized to give the product as a blue powder (5.4 mg, 5.7 µmol, 57 % yield).

MS (MALDI-TOF): $[C_{45}H_{60}N_3O_{10}S_3]^+$ calcd 898.3, found 898.9. ^1H-NMR (300 MHz, DMSO-d6): 8.36 (t, 2H, CH), 7.80 (s, 2H, Ar-H), 7.61 (d, 2H, Ar-H), 7.31 (dd, 2H, Ar-H), 6.59 (t, 1H, CH), 6.34 (t, 2H,

CH), 4.08 (m, 4H, N-CH_2), 2.03 – 1.95 (m, 6H, 3CH_2), 1.84 (s, 3H, 3CH), 1.80-1.69 (m, 2H, CH_2), 1.68 (s, 12H, C-($\underline{CH_3}$)$_2$), 1.56 (s, 6H, 3CH_2), 1.46 (m, 2H, CH_2), 1.23 (s, 6H, 3CH_2) ppm.

*Cy5-Ad$_2$ (compound **7**)*

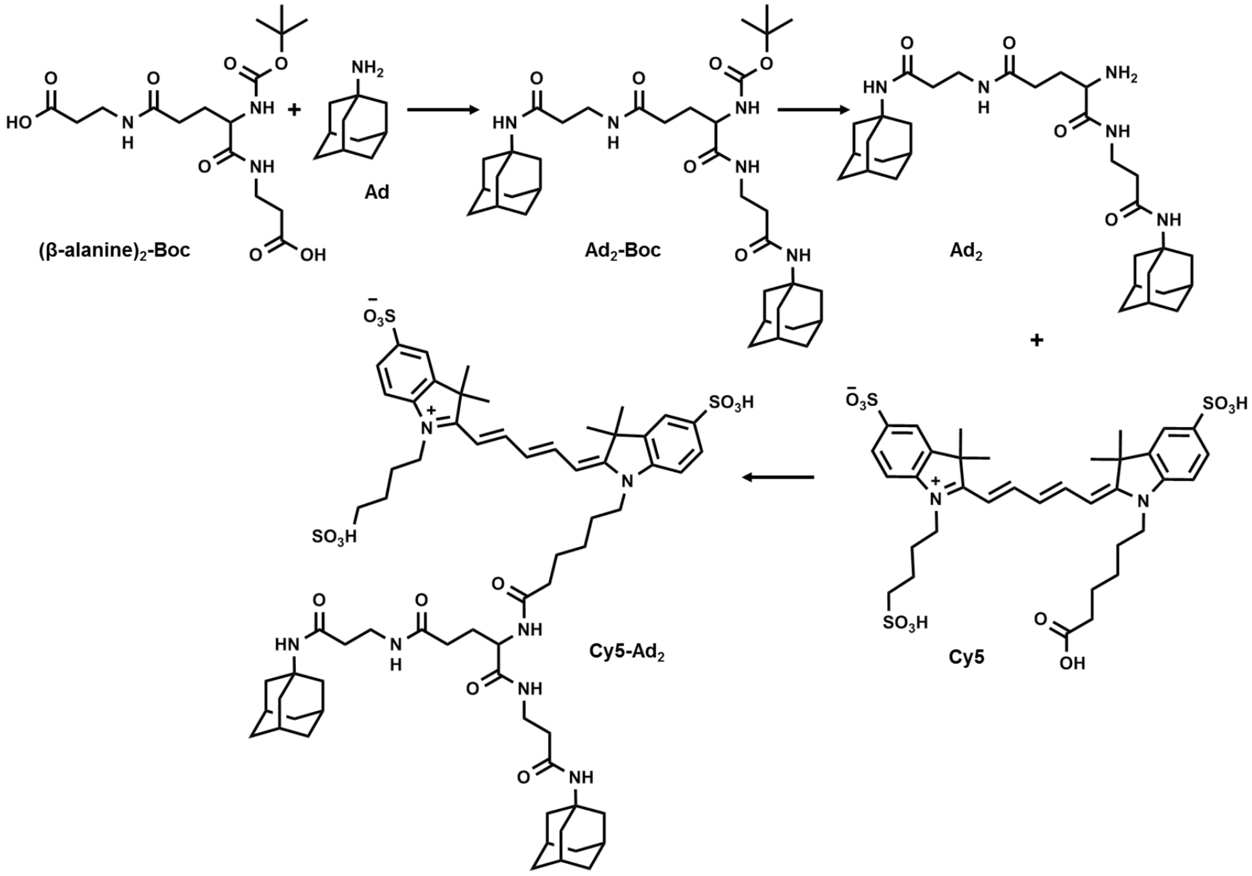

Figure S7: Synthesis of Cy5-Ad$_2$ (**7**) by conjugating Cy5-(SO_3)Sulfonate-(SO_3)COOH (Cy5, **8**) with bis-adamantane (Ad$_2$). The two Ad compounds are connected to each other via a previously synthesized β-alanine spacer.[7]

Cy5-Ad$_2$ was synthesized in multiple steps using standard peptide coupling chemistry. Boc-Glu-(β-Ala)$_2$ (101 mg, 0.26 mmol), synthesized as described before,[8] PyBOP (676 mg, 1.3 mmol) and DIPEA (530 µL, 3 mmol) were dissolved in 5 mL dry DMF. After 5 min, adamantan-1-amine hydrochloride (244 mg, 1.3 mmol) was added and the mixture was stirred for 3 h at room temperature. The product was purified by column chromatography (CH_2Cl_2:MeOH 9:1) and the product containing fractions were collected and concentrated under vacuo. The obtained

compound was dissolved in 4 mL TFA:DCM (1:4) and stirred overnight at RT. After evaporation of the solvents in vacuo, the obtained H-Glu-(β-Ala-Ad)$_2$ was dissolved in H_2O:MeCN and lyophilized to give the product as a white solid (111 mg, 0.17 mmol). Subsequently, H-Glu-(β-Ala-Ad)$_2$, (20 mg, 30 µmol) was dissolved in 2 mL dry DMF and Cy5-(SO_3)Sulfonate-(SO_3)COOH (**8**) (25 mg, 30 µmol), PyBOP (16 mg, 30 µmol) and DIPEA (17 µL, 100 µmol) were added. After stirring overnight at RT, 2 mL of 0.1 % TFA in H_2O was added to purify the product directly by preparative HPLC. The product containing fractions were collected and lyophilized to give Cy5-Ad$_2$ as a blue solid (9.0 mg, 6.9 µmol, 2.6 %).

MS (MALDI-TOF): $[C_{66}H_{92}N_7O_{14}S_3]^+$ calcd 1303.7, found 1303.9. ^{1}H-NMR (300 MHz, DMSO-d6): 8.35 (t, 2H), 7.80 (s, 2H), 7.60 (d, 2H), 7.31 (dd, 2H), 6.58 (t, 1H), 6.34 (m, 2H), 4.07 (m, 4H), 2.0-1.0 (m, 10H), 1.84 (s, 6H), 1.68 (s, 12H). ^{1}H NMR (300 MHz, DMSO-D6): 8.35 (t, 2H, CH), 7.80 (s, 4H, Ar-H), 7,60 (d, 2H, Ar-H), 7.33 (d, 2H, Ar-H), 6.58 (t, 1H, CH), 6.42 (d, 1H, CH), 6.31 (d, 1H, CH), 4.07 (m, 4H, N-CH_2), 3.36 - 3.15 (m, 5H, CH and 2$CH_{2,}$ semi covered under solvent peak), 2.29-2.02 (m, 6H, 3CH_2), 2.01 – 1.19 (m, 10H, 5CH_2), 1.88 (s, 6H, 6CH), 1.80-1.69 (m, 4H, 2CH_2) 1.68 (s, 12H, C-($\underline{CH_3}$)$_2$), 1.57 (s, 12H, 6CH_2), 1.23 (s, 12H, 6CH_2) ppm.

*Synthesis of Cy5-CD (compound **5**)*

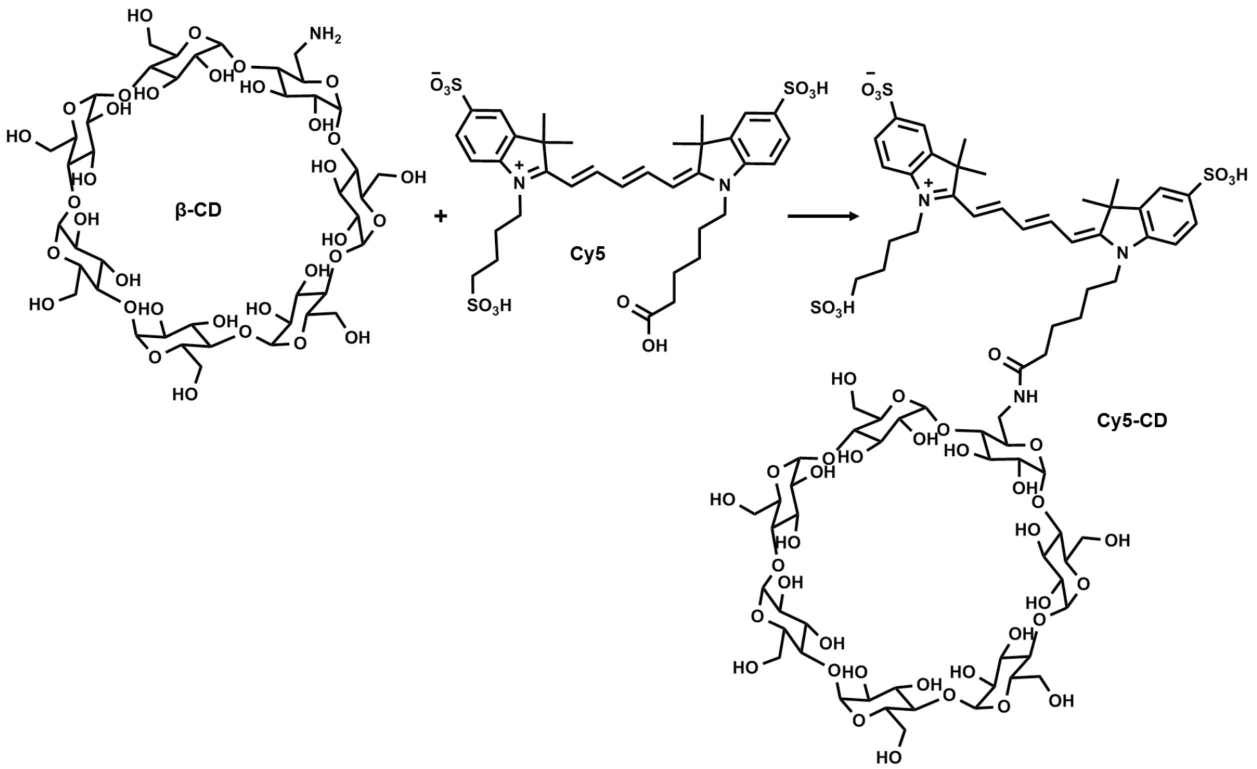

Figure S8: Conjugation of β-CD with Cy5-(SO_3)Sulfonate-(SO_3)COOH (Cy5, **8**).

Cy5-(SO_3)Sulfonate-(SO_3)COOH (**8**) (4.2 mg, 5 µmol), 6-monodeoxy-6-monoamino-β-cyclodextrin (5.9 mg, 5 µmol), and PyBOP (5.2 mg, 10 µmol) were dissolved in 1 mL dry DMSO. DIPEA (3.5 µL, 20 µmol) was added and the mixture was stirred overnight at RT. The products were precipitated in CH_2Cl_2 and purified by preparative HPLC. The product fraction was lyophilized to give a blue powder (5.2 mg, 2.7 µmol, 53%).

MS (MALDI-TOF): $[C_{77}H_{113}N_3O_{44}S_3]^+$ calcd 1880.8, found 1882.0. ^{1}H NMR (300 MHz, D_2O): 8.03 (m, 2H, CH), 7.79-7.74 (m, 4H, Ar-H), 7.29 (t, 2H, Ar-H), 6.6-6.2 (m, 3H, CH), 4.97 (m, 7H, O-CH-O-CH of β-CD), 4.05 (m, 4H, N-CH_2), 3.90-3.49 (m, 42H, all other β-CD protons), 2.91 (t, 2H, CH_2-COOH), 2.17 (t, 2H, CH_2-SO_3), 2.0-1.0 (m, 10H, 5CH_2), 1.65 (s, 12H, C-$(CH_3)_2$) ppm.

Analysis of the CD-polymers

The grafting efficiency of β-CD and the fluorophores was determined by a combination of ^{1}H-NMR and UV/Vis absorption measurements. The grafting of the β-CD was determined by ^{1}H-

NMR, by integrating the polymer peaks at 1.38 - 1.00 ppm (both methyl and CH_2 moieties) and the β-CD peaks at 5.1 ppm (anomeric carbon CH) and 4.00 - 3.50 ppm (all other β-CD protons). The integral of the peaks corresponding to the polymer was then set at 8 (Suplementary Fig. S10B). The obtained integral for the β-CD peaks at 5.1 ppm and 4.00 - 3.50 ppm were divided by 7 and 42 respectively (in theory, if one β-CD per subunit would have been present, their integrals would have been 7 and 42). The resulting ratio is the approximated percentage of β-CD per subunit. Both ratios obtained for the peak at 5.0 and 4.0 - 3.5 ppm were averaged and then multiplied by the number of subunits in the polymer.

To determine the number of fluorophores per polymer, first the fluorophore concentration of the sample was calculated by measuring the absorbance at 650 nm (Cy5) or at 550 nm (Cy3) and applying the Beer-Lambert law (equation (1)). Then the concentration of the fluorophore was correlated with the calculated concentration of the polymer, based on its estimated molecular weight.

$$A = l \cdot \varepsilon \cdot C \qquad \text{(eq.1)}$$

Where: A = absorbance, l = path length in cm, ε = absorption coefficient, C = Molar concentration

The molecular weight (MW) of the polymer was first estimated by adding together: the starting MW of the polymer (6,000 or 60,000 g/mol), the MW of β-CD (1133 g/mol) times the numbers of β-CD per polymer (0, 10, or 72), and the MW of H_2O (18 g/mol) times the number of carboxylates per polymer (78.0, 67.5, or 705.5). Then the number of fluorophores per polymer was calculated and the resulting number of fluorophore per polymer (0.4, 0.5, or 1.5) times the MW of the fluorophore was added to obtain the final MW of the polymers

The hydrodynamic radii of the polymers were determined using dynamic light scattering (DLS) and diffusion-ordered NMR spectroscopy (DOSY). Based on the diffusion constants, the hydrodynamic radii could be calculated using the Stokes-Einstein equation (Equation (2)).

$$D = \frac{k_B T}{6\pi\eta r} \qquad \text{(eq. 2)}$$

Where: D = diffusion constant, K_B = boltzmann's constant, T = absolute temperature, η = dynamic viscosity of the medium and r = radius of the particles /compound.

Cell culture

Human MDAMB231 cells, transfected with human CXCR4 conjugated GFP (MDAMB231 X4), were kindly provided by Dr. Gary Luker (Center for Molecular Imaging, University of Michigan, USA).[5] Native MDAMB231 cells with basal CXCR4 expression were used as control.[6] Cells were maintained in Dulbecco's minimum essential medium (DMEM) enriched with 10% fetal bovine serum and 5 mL Penicillin/Streptomycin (1,000 units/mL Penicillin; 1,000 µg/mL Streptomycin) (all Life Technologies Inc.). Cell lines were cultured and maintained under standard conditions (37 ^{o}C and 5% CO_2).

Confocal microscopy

One day prior the experiment, cells were trypsinized, seeded onto culture dishes with glass insert (ø35mm glass bottom dishes No. 15, poly-d-lysine coated, γ-Irradiated, MatTek corporation) and incubated overnight in 2 mL DMEM. After incubation of the compounds (see below for the conditions of each experiment), live cell images were taken on a Leica SP5 or SP8 WLL confocal microscope under 63x magnification. The intrinsic GFP signal in the MDAMB231 X4 cells was measured with excitation at 488 nm and emission was collected at 500-525 nm. Cy3 fluorescence was measured with excitation at 514 nm, emission was collected at 550-570 nm. Cy5 fluorescence was measured with excitation at 633 nm, emission was collected at 650-700 nm. Any Hoechst 33342 fluorescence was measured using 405 nm excitation and emission was collected at 420-470 nm. Images and signal quantifications were obtained using Leica Application Suite software, by applying the polygon function and calculating the average gray value/m^2 for each cell. For quantifications; background signal (amount of grey value/m^2 when no compounds are added) was subtracted from the fluorescence signal obtained for the samples.

Comparison of multivalent and monovalent functionalization

To study the differences in binding of multivalent- and monovalent β-CD compounds to **Ac-TZ14011-Ad** functionalized cells, adhering MDAMB231 X4 cells were functionalized with either

$Cy5_{0.5}CD_{10}PIBMA_{39}$, **$Cy5_{0.4}PIBMA_{39}$**, or **Cy5-CD** (according described procedure; 'Functionalization of cells', see main manuscript), in such a way that the polymer concentration was 1 µM (**$Cy5_{0.5}CD_{10}PIBMA_{39}$** and **$Cy5_{0.4}PIBMA_{39}$**) and the β-CD concentration was 10 µM (**$Cy5_{0.5}CD_{10}PIBMA_{39}$** and **Cy5-CD**). After washing twice with PBS, confocal images were taken. For quantification, for each condition of 25 cells the average grey value/m^2 was measured (Suplementary Fig. S14 and S15).

*Competition between **$Cy5_{0.5}CD_{10}PIBMA_{39}$** and **$Cy3_{1.5}CD_{72}PIBMA_{389}$** (monitored using microscopy)*

To compare the binding strength between **$Cy5_{0.5}CD_{10}PIBMA_{39}$** and **$Cy3_{1.5}CD_{72}PIBMA_{389}$** MDA231 X4 cells were functionalized with either **$Cy5_{0.5}CD_{10}PIBMA_{39}$** (10 µM final β-CD concentration), **$Cy3_{1.5}CD_{72}PIBMA_{389}$** (10 µM final β-CD concentration), or a mixture of **$Cy5_{0.5}CD_{10}PIBMA_{39}$** and **$Cy3_{1.5}CD_{72}PIBMA_{389}$** (20 uM final β-CD concentration; 10 uM each). After washing twice with PBS confocal images were taken of the different samples. To determine the degree of binding of the polymers under the different conditions the average grey value/m^2 was determined for 25 cells in the individual samples (Suplementary Fig. S17)

To follow the competition over time, adherent MDAMB231 X4 cells were functionalized with either **$Cy5_{0.5}CD_{10}PIBMA_{39}$** or **$Cy3_{1.5}CD_{72}PIBMA_{389}$** (10 µM final β-CD concentration). Subsequently, they were imaged at RT (without washing first) and either **$Cy5_{0.5}CD_{10}PIBMA_{39}$** or **$Cy3_{1.5}CD_{72}PIBMA_{389}$** (10 µM final β-CD concentration) were added. The change in fluorescence, was followed for 18 minutes, while taking images each minute (Supplementary Fig. S18 and S19).

*Replacement between **$Cy5_{0.4}PIBMA_{39}$**, **$Cy5_{0.5}CD_{10}PIBMA_{39}$** and **$Cy3_{1.5}CD_{72}PIBMA_{389}$** (monitored using microscopy)*

The replacement between **$Cy5_{0.5}CD_{10}PIBMA_{39}$** and **$Cy3_{1.5}CD_{72}PIBMA_{389}$**, was performed according the same procedure as described for the competition experiment followed over time. Only now the excess of binding polymer was washed away with PBS (2 x 1 mL) before imaging. As a control, besides the respective polymers, also only medium was added. The change in

fluorescence was followed for 18 minutes, while taking images each minute (Supplementary Fig. S18 and S19).

The replacement of the non-host containing **$Cy5_{0.4}PIBMA_{39}$** polymer by **$Cy3_{1.5}CD_{72}PIBMA_{389}$** and the reverse were performed according the same procedure as described above. The cells were incubated with **$Cy5_{0.4}PIBMA_{39}$** (1 μM polymer) or **$Cy3_{1.5}CD_{72}PIBMA_{389}$** (10 μM final β-CD concentration) and during imaging **$Cy3_{1.5}CD_{72}PIBMA_{389}$** (10 μM final β-CD concentration) or **$Cy5_{0.4}PIBMA_{39}$** (1 μM polymer) was added (Supplementary Fig. S18 and S19).

Functionalization of human stem cells

Human fetal heart stem cells, with CXCR4 expression, (17 weeks after gestation) were grown in M199 -/- on gelatin-coated glass-bottom dishes (100,000 cells per dish). These cells were functionalized with **Ac-TZ14011-Ad** and **$Cy3_{1.5}CD_{72}PIBMA_{389}$** according described procedure (see 'functionalization of cells', main manuscript). Subsequently, they were carefully washed with colorless DMEM (2 x 1 mL) and analyzed by confocal microscopy (Supplementary Fig. S22).

Flow cytometry

MDAMB231 X4 cells were trypsinized (using 0.5% trypsin/EDTA, BD Biosciences) and counted. Hereafter cells were divided into aliquots (300,000 cells per tube), centrifuged for three minutes (3000 x *g*, 4 ^{o}C), and the supernatant was decanted. After incubation of the compounds (see experimental description for detailed conditions) flow cytometry measurements were performed on a BD FacsCanto™ II. Live cells were gated using forward scatter and side scatter, and 10,000 viable cells were analyzed for each sample. Cy5 fluorescence was measured on the APC channel and Cy5.5 fluorescence (for Ac-TZ14011-MSAP) was measured on the APC-Cy7 channel. For quantification; background signal (amount of fluorescence when no functionalizations were added) was subtracted from the fluorescence signal obtained for all samples.

Determination of the receptor affinity of Ac-TZ14011-Ad

The affinity (K_D) of **Ac-TZ14011-Ad** was calculated from flow cytometry measurements, using an earlier described procedure.[8] In short: Different concentrations of Ac-TZ14011-Ad, ranging between 0.5 – 15,000 nM in 120 µL PBS, were added to MDAMB231 X4 cells in the presence of Ac-TZ14011-MSAP (250 nM), a compound with well-defined receptor affinity.[8] After one hour of incubation on ice, the cells were washed two times with PBS (centrifuged 3 min, 3000 x *g*, 4 °C), and resuspended in 300 µL PBS. The fluorescence of the reference compound was measured as described in the flow cytometry section. All experiments were performed in duplicate (n = 2). The mean fluorescence was normalized and fitted with equations in the GraphPad Prism 6 software (Supplementary Fig. S11). The K_D values were calculated using the "Binding-Competitive, One site-Fit Ki" nonlinear regression equation (Equation (3) and (4)), where the used K_D value of Ac-TZ14011-MSAP (187 nM) has previously been reported.[8]

$$\log IC_{50} = \log(10^{\log K_D} * (1 + \frac{[MSAP]}{[K_{D,MSAP}]})) \qquad (eq. 3)$$

$$y = \frac{1}{1 + 10^{x - \log IC_{50}}} \qquad (eq. 4)$$

IC_{50} = concentration of the competitor that results in 50% binding, K_D = dissociation constant of the competitor in nM, [MSAP] = concentration of Ac-TZ14011-MSAP (250 nM), $K_{D,MSAP}$ = dissociation constant of Ac-TZ14011-MSAP (187 nM), y = normalized fluorescence, x = concentration of peptide-Ad in nM.

*Competition between **$Cy5_{0.5}CD_{10}PIBMA_{39}$ and $Cy3_{1.5}CD_{72}PIBMA_{389}$** (monitored using flow cytometry)*

To analyze the degree of binding of **$Cy5_{0.5}CD_{10}PIBMA_{39}$** to **Ac-TZ14011-Ad** functionalized MDA231 X4 cells in the presence of a competitor or DMEM, MDAMB231 X4 cells were trypsinized and divided into aliquots (300,000 cells per tube). The aliquots were centrifuged for four minutes (3000 x *g*, 4 ^{o}C), the supernatant was decanted and the cells were resuspended in 50 µL **Ac-TZ14011-Ad** (10 µM) in DMEM. After 1 h incubation at 0 ^{o}C the cells were centrifuged again for four minutes (3000 x *g*, 4 ^{o}C), the supernatant was decanted and 100 µL of either

$Cy5_{0.5}CD_{10}PIBMA_{39}$ (10 μM β-CD) or a mixture of **$Cy5_{0.5}CD_{10}PIBMA_{39}$** and **$Cy3_{1.5}CD_{72}PIBMA_{389}$** (each 10 μM β-CD) in DMEM was added. Another hour at 0 ^{o}C followed. The cells were then washed twice with PBS (centrifuged 3 min, 3000 x g, 4 ^{o}C), resuspended in 150 μL PBS and the intensity of Cy5 fluorescence related to the cells was measured by flow cytometry. Due to presence of GFP, the intensity of Cy3 fluorescence could not be monitored. This experiment was performed in two-fold (Supplementary Fig. S17).

Replacement between $Cy5_{0.5}CD_{10}PIBMA_{39}$ and $Cy3_{1.5}CD_{72}PIBMA_{389}$ quantified by radioactivity

Radiolabeling of **$Cy5_{0.5}CD_{10}PIBMA_{39}$** and **$Cy3_{1.5}CD_{72}PIBMA_{389}$** with technetium-99m was performed as follows: **$Cy5_{0.5}CD_{10}PIBMA_{39}$** (75 μL, 43.8 nmol β-CD) or **$Cy3_{1.5}CD_{72}PIBMA_{389}$** (75 μL, 38 nmol β-CD), was mixed with $SnCl_2.2H_2O$ (4 μL of 1 mg/mL saline solution, 17.7 nmol , Technescan PYP Kit, Mallinckrodt Medical B.V., Petten, The Netherlands) and freshly eluted ^{99m}Tc-Na-pertechnetate (200 μL, Technekow, Mallinckrodt Medical B.V.) and gently stirred for 1 h at RT. Thereafter, the reaction mixture was purified from free ^{99m}Tc by size exclusion chromatography using sterile PBS as mobile phase on Sephadex™ G-25 desalting columns (PD-10, GE Healthcare Europe GmbH, Freiburg, Germany). Fractions containing radiolabeled **$Cy5_{0.5}CD_{10}PIBMA_{39}$** (^{99m}Tc-$Cy5_{0.5}CD_{10}PIBMA_{39}$) or **$Cy3_{1.5}CD_{72}PIBMA_{389}$** (^{99m}Tc-$Cy3_{1.5}CD_{72}PIBMA_{389}$) were collected and directly applied in the assembly studies at a final concentration of 13 μM β-CD).

MDAMB231 X4 cells were harvested, counted and diluted to 80,000 cells/mL using DMEM. Of this solution, 200 μL (16,000 cells) fractions were transferred to polystyrene tube (FACS) and the cells were cooled on ice for 15 minutes. Subsequently, **Ac-Tz14011-Ad** (100 μL, 11 μM) was added and another incubation of 15 minutes followed. Thereafter, the mixture was centrifuged (5 min, 1250 x *g,* 4 ^{o}C) and the supernatant was aspirated. The cells were resuspended in 200 μL DMEM and either **^{99m}Tc-$Cy5_{0.5}CD_{10}PIBMA_{39}$, ^{99m}Tc-$Cy3_{1.5}CD_{72}PIBMA_{389}$, $Cy5_{0.5}CD_{10}PIBMA_{39}$**, or **$Cy3_{1.5}CD_{72}PIBMA_{389}$** (100 μL, 13 μM β-CD) was added, and incubation followed for 15 min at 0 ^{o}C. Thereafter, the mixture was centrifuged as described before. After aspiration of the supernatant and suspension of the cells in 200 μL DMEM, either **$Cy5_{0.5}CD_{10}PIBMA_{39}$, $Cy3_{1.5}CD_{72}PIBMA_{389}$, ^{99m}Tc-$Cy5_{0.5}CD_{10}PIBMA_{39}$**, or **^{99m}Tc-$Cy3_{1.5}CD_{72}PIBMA_{389}$**

(100 µL, 13 µM β-CD) was added and the samples were shortly vortexed. To challenge the strength of the binding of the fluorescent β-CD polymers to MDAMB231 X4 cells, incubation of 1 h at 0 ^{o}C followed. After centrifugation of the cells and aspiration of the supernatant both were counted for radioactivity in a dose-calibrator or gamma counter to assess the amount of cellular-bound ^{99m}Tc-Cy_n-CD_x-$PIBMA_y$ activity. Data was expressed as the mean % (±SD, n = 6) of the total amount of ^{99m}Tc-Cy_n-CD_x-$PIBMA_y$ activity added to the cells (Supplementary Fig. S16).

Cell viability assay

To determine the effect of polymer functionalization on the cell viability, after cell functionalization a MTT test was performed according to a described procedure.[9,10] MDAMB231 X4 cells (16,000 cells per tube) in 200 µL DMEM were cooled on ice for 15 minutes. **Ac-Tz14011-Ad** (100 µL, 10 µM) was added and incubation of 15 minutes followed. Thereafter, the mixture was centrifuged (5 min, 1250 x *g,* 4 ^{o}C) and the supernatant was aspirated. The cells were resuspended in 200 µL DMEM and variable concentrations of either **$Cy5_{0.5}CD_{10}PIBMA_{39}$** or **$Cy3_{1.5}CD_{10}PIBMA_{389}$** (100 µL, 0-16 µM final β-CD concentration) were added. After 15 min incubation on ice, the cells were washed twice with PBS (centrifuged 5 min, 1250 x g, 4 ^{o}C), resuspended in 400 µL DMEM (16,000 cells per tube) and transferred in 200 µL duplicates to a 96-wells plate (Cellstar®, Greiner Bio-One, Alphen a/d Rijn, The Netherlands) with 8,000 cells per well. After 24 h incubation at 37 ^{o}C, 20 µL (0.1 mg) of a MTT (3-(4,5-dimethylthiazol-2-yl)-2,5-diphenyltetrazolium bromide) solution in PBS (5 mg/mL) was added to each well, followed by gently shaking the plate and then by incubation for 4 h at 37 ^{o}C. Thereafter, cells were optically checked under a light microscope to determine the uptake and colouring of MTT. The medium was carefully removed and 100 µL of DMSO was added to each well to extract the insoluble formazan product from the cells. After 10-15 minutes at 37 ^{o}C, the uptake of MTT in each well was determined by measuring the absorbance at 545 nm. Measurements were normalized to those of untreated cells which were kept on ice during the entire procedure of polymer functionalization (Supplementary Fig. S13)

Results

Synthesis of Cy5-CD, Cy5-Ad and Cy5Ad$_2$

To compare cell functionalization with multivalent β-CD groups (**Cy5$_{0.5}$CD$_{10}$PIBMA$_{39}$, Cy3$_{1.5}$CD$_{72}$PIBMA$_{389}$**) to monovalent β-CD, a Cy5 fluorophore was conjugated to β-CD via amide bond formation to give **Cy5-CD** (52% yield). By the same synthesis strategy **Cy5-Ad** was synthesized (57% yield), as mode to investigate if polymer coated cells could further be functionalized via de CD-Ad host-guest interaction. To strengthen this interaction also a **Cy5-Ad$_2$** was synthesized (2.6% yield). Both Ad molecules were connected to the Cy5 and each other via a short spacer consisting of 2 beta alanines,[7] again functionalization could be established via multiple amide bond formations.

Functionalization: mono vs multivalent β-CD hosts

Comparing the fluorescence signal intensities after functionalization with **Cy5-CD** (monovalent host) or **Cy5$_{0.5}$CD$_{10}$PIBMA$_{39}$** (multivalent host), reveals substantial more binding to Ad functionalized cell surfaces by **Cy5$_{0.5}$CD$_{10}$PIBMA$_{39}$** (supplementary Fig. S14 and S15). As a control the same functionalization was also performed with **Cy5$_{0.4}$PIBMA** (Supplementary Fig S7). While **Cy5$_{0.4}$PIBMA$_{39}$** binds slightly more to the cell surface compared to **Cy5-CD**, it are the multiple CD groups on **Cy5$_{0.5}$CD$_{10}$PIBMA$_{39}$** that considerably increases the amount of binding.

Radiolabeling of the polymers

Cy5$_{0.5}$CD$_{10}$PIBMA$_{39}$, and **Cy3$_{1.5}$CD$_{72}$PIBMA$_{389}$** were radiolabeled with technetium-99m according to a modification of a previously described labeling technique (described above).[11] We propose that the carboxylates, formed after hydrolysis, and possibly also the side chains of β-CD could function as chelate for the technetium-99m ions after reduction with $SnCl_2$. Since no significant

release of radioactivity from the polymer was observed until 24 hours after radiolabeling, the interaction was stable under the *in vitro* conditions here applied.

Exchange and competition between CD_nPIBMA_m polymers

To monitor the strength and reversibility of the PIBMA surface modifications, exchange experiments were performed between radiolabeled and non-radiolabeled CD_nPIBMA_m polymers. After functionalizing MDAMB231 X4 cells with either **^{99m}Tc-Cy5$_{0.5}$CD$_{10}$PIBMA$_{39}$** or **^{99m}Tc-Cy3$_{1.5}$CD$_{72}$PIBMA$_{389}$**, both polymers remained attached to the cell membrane for at least 1 hour in PBS. However, the use of either **Cy5$_{0.5}$CD$_{10}$PIBMA$_{39}$** or **Cy3$_{1.5}$CD$_{72}$PIBMA$_{389}$** as competitor resulted in a decrease in signal (Supplementary Fig. S16A and C). Binding of the competitors was confirmed when the experiment was performed with non-radioactive **Cy5$_{0.5}$CD$_{10}$PIBMA$_{39}$** or **Cy3$_{1.5}$CD$_{72}$PIBMA$_{389}$** functionalized MDAMB231 X4 cells and using ^{99m}Tc-labeled competitors, indicating an exchange between the two polymers (Supplementary Fig. S16B and D).

The polymer exchange was visually confirmed using confocal microscopy experiments performed over time (0 – 18 min), at the same β-CD concentrations (Supplementary Fig. S18B and S19B). Supplementary Fig S18B shows a decrease in **Cy5$_{0.5}$CD$_{10}$PIBMA$_{39}$** signal, while at the same time the **Cy3$_{1.5}$CD$_{72}$PIBMA$_{389}$** signal increases, indicating an exchange between the two polymers. In these images the signal of **Cy5$_{0.5}$CD$_{10}$PIBMA$_{39}$** is nearly gone after 18 minutes. In the absence of **Cy3$_{1.5}$CD$_{72}$PIBMA$_{389}$**, this decrease in **Cy5$_{0.5}$CD$_{10}$PIBMA$_{39}$** was not observed (Supplementary Fig. S18A). This confocal experiment was repeated, using **Cy5$_{0.4}$PIBMA$_{39}$** (Supplementary Fig. S18D). The signal of **Cy5$_{0.4}$PIBMA$_{39}$** did not decrease over time, indicating a clear difference of interaction compared to **Cy5$_{0.5}$CD$_{10}$PIBMA$_{39}$** (with CD-moieties). In the presence of **Cy5$_{0.4}$PIBMA$_{39}$** the signal of **Cy3$_{1.5}$CD$_{72}$PIBMA$_{389}$** still increased over time, although it was less intense (Supplementary Fig. S18D). To validate these findings the experiment was also performed in reversed order (**Cy3$_{1.5}$CD$_{72}$PIBMA$_{389}$** as initial binder and **Cy5$_{0.4}$PIBMA$_{39}$** as competitor (Supplementary Fig. S19D)). Here initial non-specific binding of **Cy5$_{0.4}$PIBMA$_{39}$** occurred, but no further increase in binding could be observed over time. The

$Cy3_{1.5}CD_{72}PIBMA_{389}$ signal only showed a drop following initial non-specific **$Cy5_{0.4}PIBMA_{39}$** binding. When the cells are only washed and no Cy5 polymer is added, no such decrease in Cy3 signal was observed (Supplementary Fig. S19A). These results suggest that **$Cy5_{0.4}PIBMA_{39}$** binds on a different location and via a different type of interaction than **$Cy3_{1.5}CD_{72}PIBMA_{389}$** (non-specific vs. specific host-guest). These same results also indicate that the non-specific binding of **$Cy5_{0.4}PIBMA_{39}$** hinders **$Cy3_{1.5}CD_{72}PIBMA_{389}$** via electrostatic repulsion, thereby influencing the binding equilibrium.

To further explore the difference in affinity of the two CD-polymers for Ad functionalized cell surfaces, competition experiments were performed. **Ac-TZ14011-Ad** functionalized MDAMB231 X4 cells were incubated with a mixture of both polymers at equal β-CD concentration. This resulted in more **$Cy3_{1.5}CD_{72}PIBMA_{389}$** bound to the cell surface then **$Cy5_{0.5}CD_{10}PIBMA_{39}$** (Supplementary Fig. S17). According these results **$Cy3_{1.5}CD_{72}PIBMA_{389}$** seems to have higher affinity for **Ac-TZ14011-Ad** functionalized MDAMB231 X4 cells then **$Cy5_{0.5}CD_{10}PIBMA_{39}$.** Exchange was also attempted following functionalization, in the presence of an excess of the initial binder revealed that **$Cy5_{0.5}CD_{10}PIBMA_{39}$** can partly be competed off the surface by **$Cy3_{1.5}CD_{72}PIBMA_{389}$** (Supplementary Fig. S18C), while **$Cy3_{1.5}CD_{72}PIBMA_{389}$** cannot visibly be replaced by **$Cy5_{0.5}CD_{10}PIBMA_{39}$** (Supplementary Fig. S19C). This data provides a further indication that **$Cy3_{1.5}CD_{72}PIBMA_{389}$** is the better binder of the two.

Combined, these data suggest that the polymer functionalization is stable when no competition occurs, but is reversible under competitive conditions. Furthermore, the data confirms that there is a different mode of action for polymers that contain β-CD vs. those that don't.

Supplementary images

Table S1: Determined parameters of the synthesized polymers

	MW start (kDa)	Repeating units	CD/ polymer	Carboxylates/ polymer	Dye/ polymer	MW final (kDa)	Ø (DLS) (nm)	Ø (DOSY) (nm)
$Cy5_{0.4}$ $PIBMA_{39}$	6	39	0	78	0.4 Cy5	7.8	[a]	2.7
$Cy5_{0.5}CD_{10}$ $PIBMA_{39}$	6	39	10	67.5	0.5 Cy5	18.8	[a]	2.8
$Cy3_{1.5}CD_{72}$ $PIBMA_{389}$	60	389	72	704.5	1.5 Cy3	155.0	18.4	11.7

[a] Values could not be determined due to Cy5 influence.

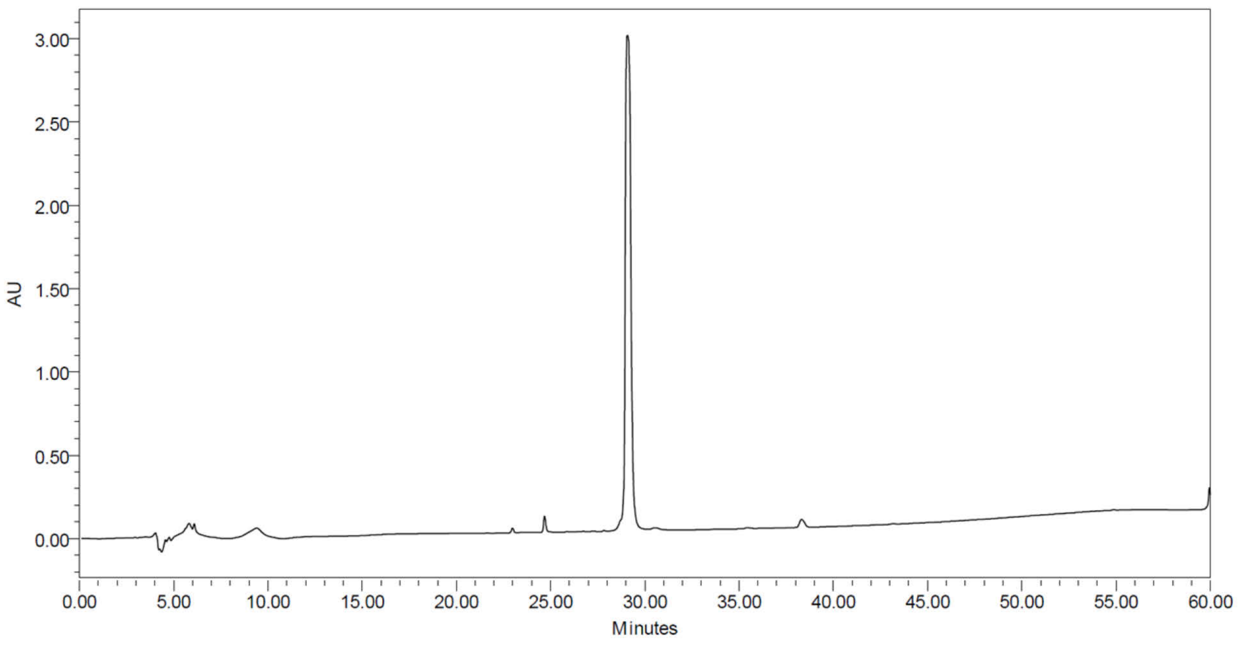

Figure S9: Analytical HPLC of **Ac-TZ14011-Ad**

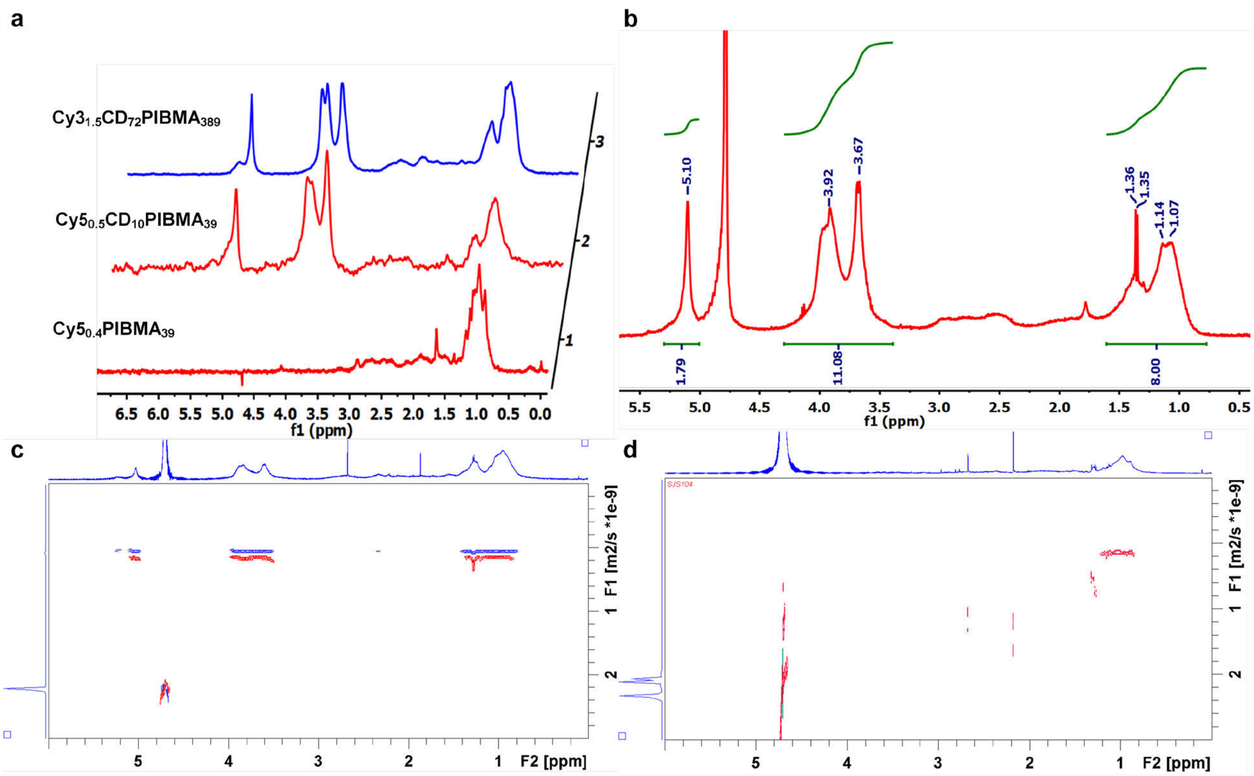

Figure S10. **a)** Diffusion-filtered NMR spectra of **Cy3$_{1.5}$CD$_{72}$PIBMA$_{389}$, Cy5$_{0.5}$CD$_{10}$PIBMA$_{39}$** and **Cy5$_{0.4}$PIBMA$_{39}$** . Represented in red the Cy5 polymers and in blue the Cy3. **b)** ^{1}H-NMR spectrum of **Cy5$_{0.5}$CD$_{10}$PIBMA$_{39}$** showing integration of the β-CD peaks at 5.1 ppm (anomeric carbon CH) and at 3.92 - 3.67 ppm (all other β-CD protons), and the polymer peaks at 1.36 - 1.07 ppm (both methyl and CH_2 moieties) The later integral was set at 8 to calculate the β-CD per polymer ratio by dividing the integrals at 5.1 and 3.92 – 3.67 ppm by 7 and 42 respectively (see ‘analysis of the polymers’). **c)** DOSY spectra of **Cy5$_{0.5}$CD$_{10}$PIBMA$_{39}$** (red) and **Cy3$_{1.5}$CD$_{72}$PIBMA$_{389}$** (blue). Vertical axis shows diffusion coefficient, with the largest molecules appearing at the top of the graph. **d)** DOSY spectrum of **Cy5$_{0.4}$PIBMA$_{39}$** .

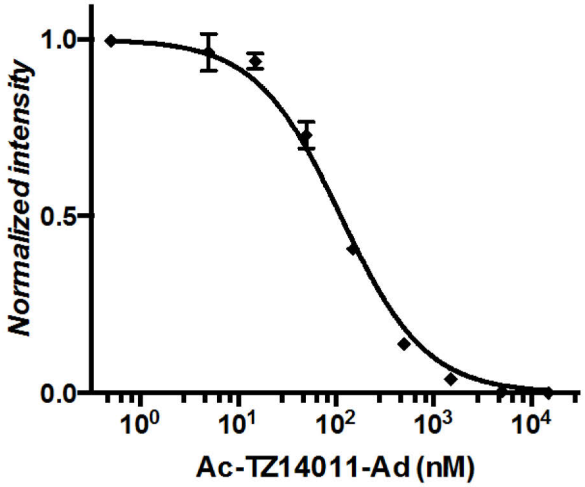

Figure S11: Competition assay curve of **Ac-TZ14011-MSAP** with **Ac-TZ14011-Ad,** acquired by flow cytometry, to determine binding constant (K_D).

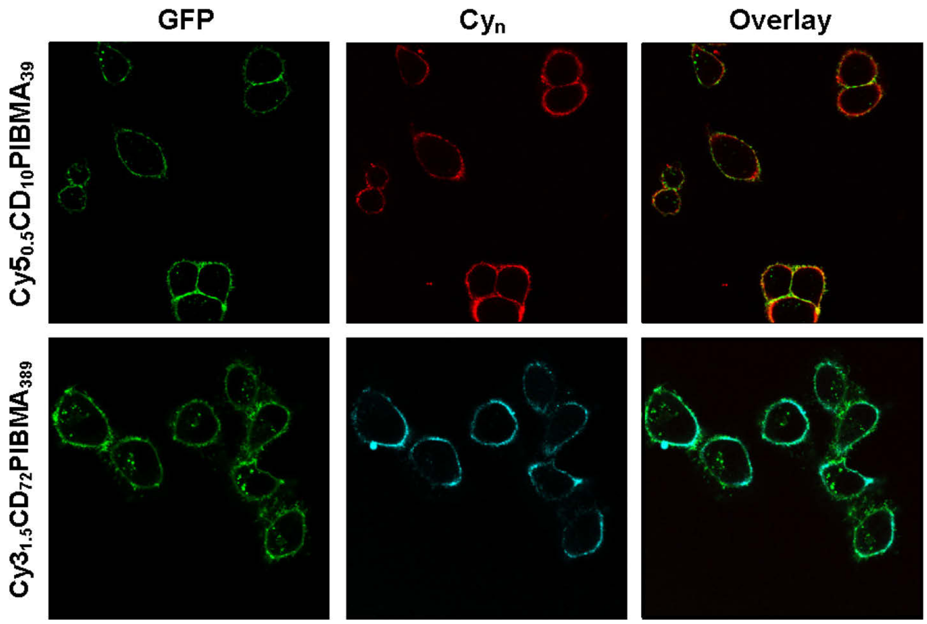

Figure S12. Viable CXCR4 overexpressing MDAMB231 X4 cells (with GFP-Tag) functionalized with either **Cy5$_{0.5}$CD$_{10}$PIBMA$_{39}$** or **Cy3$_{1.5}$CD$_{72}$PIBMA$_{389}$**. The CXCR4 receptor was first targeted with **Ac-TZ14011-Ad**, followed by functionalization with fluorescent **Cy5$_{0.5}$CD$_{10}$PIBMA$_{39}$** or **Cy3$_{1.5}$CD$_{72}$PIBMA$_{389}$** via the host-guest interaction between the β-CD molecules and the Ad functionality**.** For clarity, both the (overlay) image and the same image at the individual channels are displayed, with GFP in green, Cy5 (**Cy5$_{0.5}$CD$_{10}$PIBMA$_{39}$**) in red, and Cy3 (**Cy3$_{1.5}$CD$_{72}$PIBMA$_{389}$**) in blue.

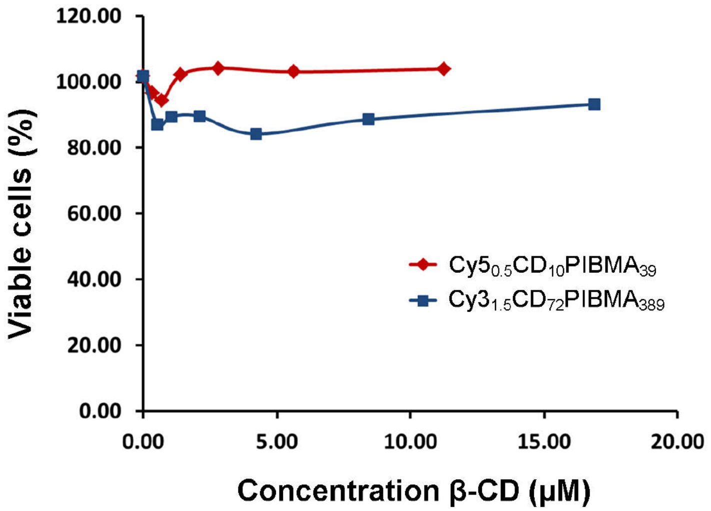

Figure S13: Viability of MDAMB231 X4 cells measured 24 h after functionalization with either **Cy5$_{0.5}$CD$_{10}$PIBMA$_{39}$** (red) or **Cy3$_{1.5}$CD$_{72}$PIBMA$_{389}$** (blue) at variable polymer concentrations (0 – 16 µM).

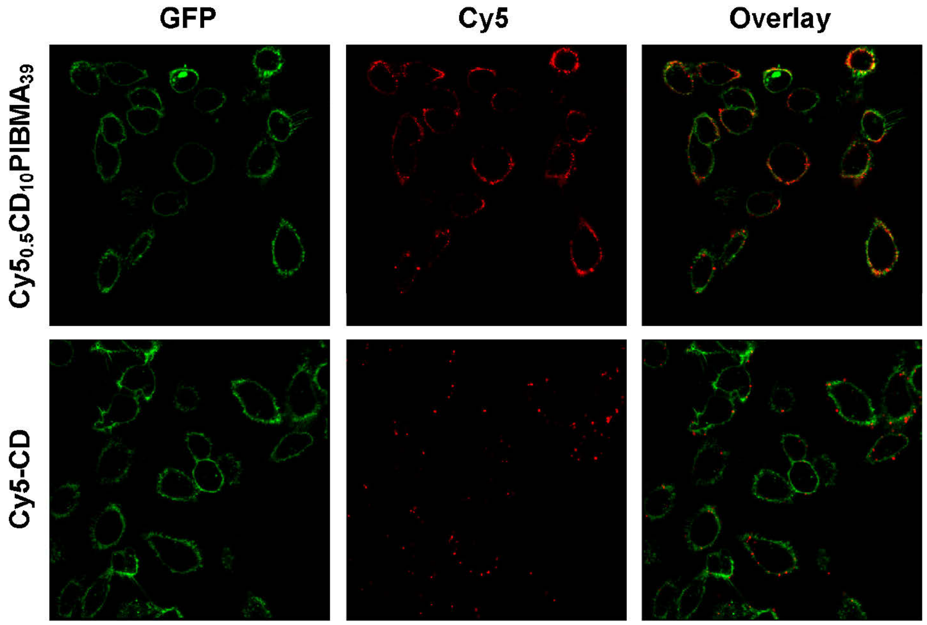

Figure S14: Comparison of multivalent and monovalent functionalization of viable cell surfaces. MDAMB231 X4 cells incubated with **Ac-TZ14011-Ad**, and subsequently with **Cy5$_{0.5}$CD$_{10}$PIBMA$_{39}$** or **Cy5-CD**. For clarity, both the (overlay) image and the same image at the individual channels are displayed, with GFP in green and Cy5 in red.

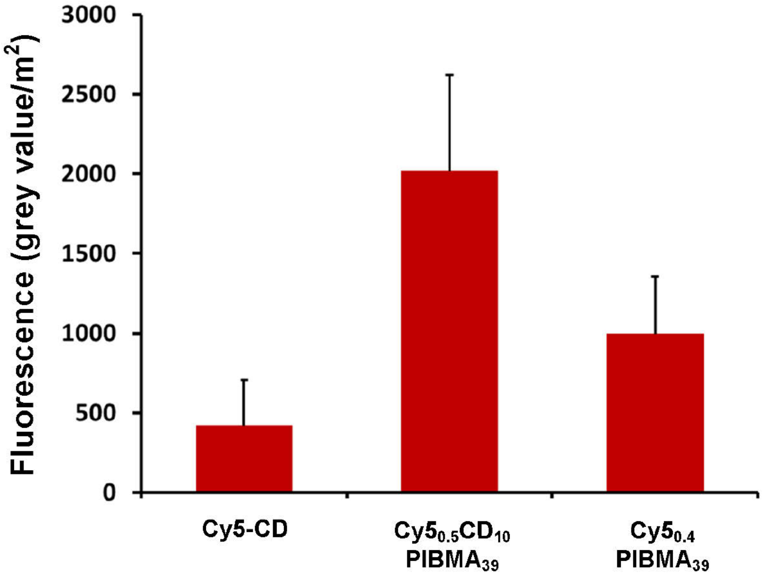

Figure S15: Comparison in binding of **Cy5-CD** (monovalent host), **$Cy5_{0.5}CD_{10}PIBMA_{39}$** (multivalent host), and **$Cy5_{0.4}PIBMA_{39}$** (no host) to **Ac-TZ14011-Ad** functionalized MDAMB231 X4 cells. The degree of binding was quantified by confocal microscopy, the graph shows the normalized data with the error bars indicating the standard deviation (25 cells analyzed for each condition).

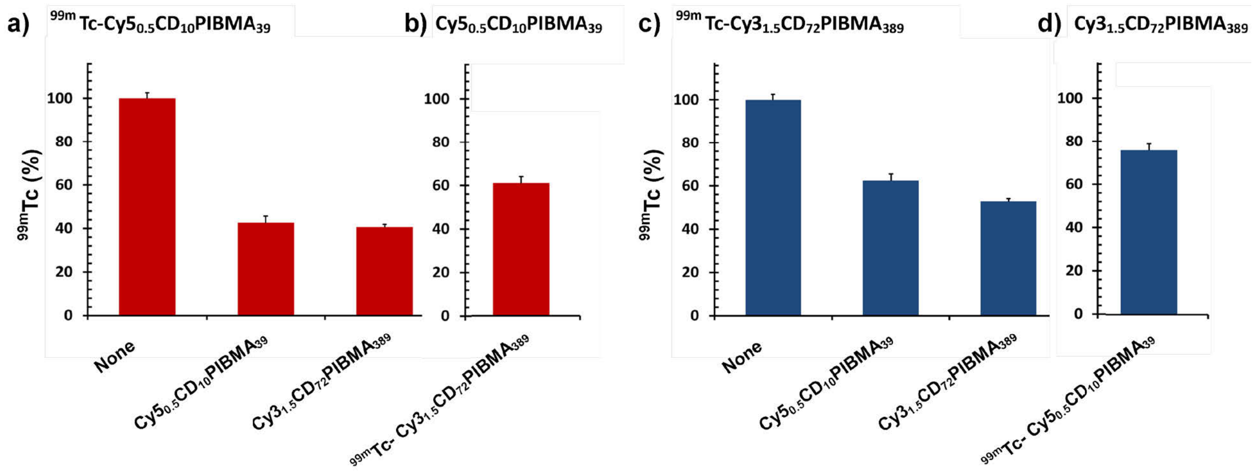

Figure S16. Exchange experiments between radiolabeled and non-radiolabeled CD_nPIBMA_m polymers on MDAMB231 cells, monitored by radioactivity after 1 h of incubation with the competitor (**a-d**). **a** - **b**) MDAMB231 X4 cells functionalized with **$Cy5_{0.5}CD_{10}PIBMA_{39}$**, either radiolabeled **(a)** or non-radiolabeled **(b). c** - **d**) MDAMB231 X4 cells functionalized with radiolabeled **$Cy3_{1.5}CD_{72}PIBMA_{389}$ (c)** or non-radiolabeled **$Cy3_{1.5}CD_{72}PIBMA_{389}$ (d).** As competitor, **$Cy5_{0.5}CD_{10}PIBMA_{39}$** or **$Cy3_{1.5}CD_{72}PIBMA_{389}$** were

added to the cells functionalized with ^{99m}Tc-CD$_n$PIBMA$_m$ polymers (**a** and **c**), showing partial replacement of the original polymer. In **b** and **d**, **^{99m}Tc-Cy5$_{0.5}$CD$_{10}$PIBMA$_{39}$** or **^{99m}Tc-Cy3$_{1.5}$CD$_{72}$PIBMA$_{389}$** were added as competitor to cells functionalized with non-radioactive CD$_n$PIBMA$_m$ polymers, showing binding of the competitor.

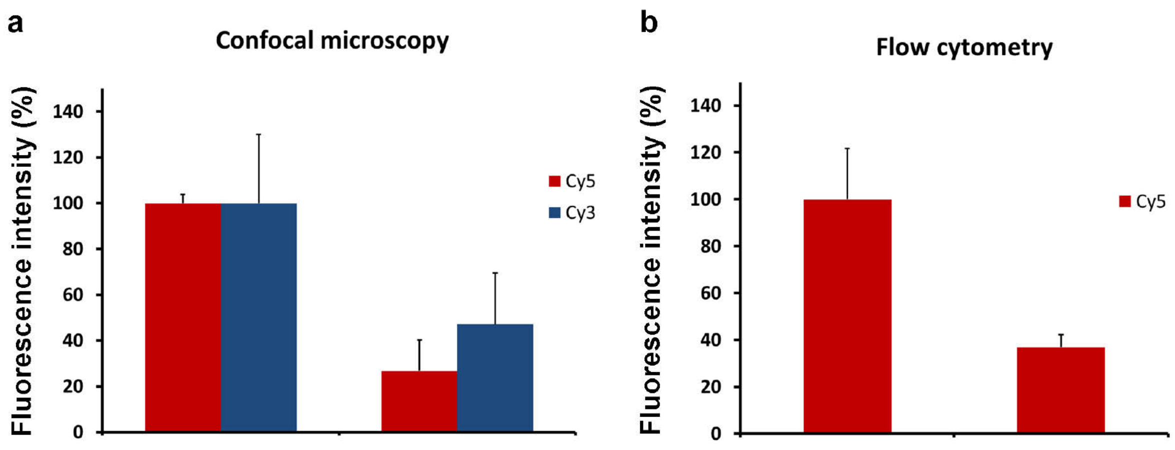

Figure S17. Fluorescence intensities obtained after incubation of **Ac-TZ14011-Ad** functionalized MDAMB231 X4 cells with a mixture of **Cy5$_{0.5}$CD$_{10}$PIBMA$_{39}$** and **Cy3$_{1.5}$CD$_{72}$PIBMA$_{389}$** (equal β-CD concentration). **a)** Fluorescence intensity of **Cy5$_{0.5}$CD$_{10}$PIBMA$_{39}$** measured by flow cytometry, showing the reduction in **Cy5$_{0.5}$CD$_{10}$PIBMA$_{39}$** binding when **Cy3$_{1.5}$CD$_{72}$PIBMA$_{389}$** was present during incubation. **b)** Fluorescence intensity of **Cy5$_{0.5}$CD$_{10}$PIBMA$_{39}$** and **Cy3$_{1.5}$CD$_{72}$PIBMA$_{389}$** measured by confocal microscopy, showing the reduction in **Cy5$_{0.5}$CD$_{10}$PIBMA$_{39}$**- (in red) and **Cy3$_{1.5}$CD$_{72}$PIBMA$_{389}$** binding (in blue) when both polymers are present during incubation. **Cy3$_{1.5}$CD$_{72}$PIBMA$_{389}$** binds stronger as the binding of **Cy5$_{0.5}$CD$_{10}$PIBMA$_{39}$** is more reduced. The error bars indicate the standard deviation (n = 2).

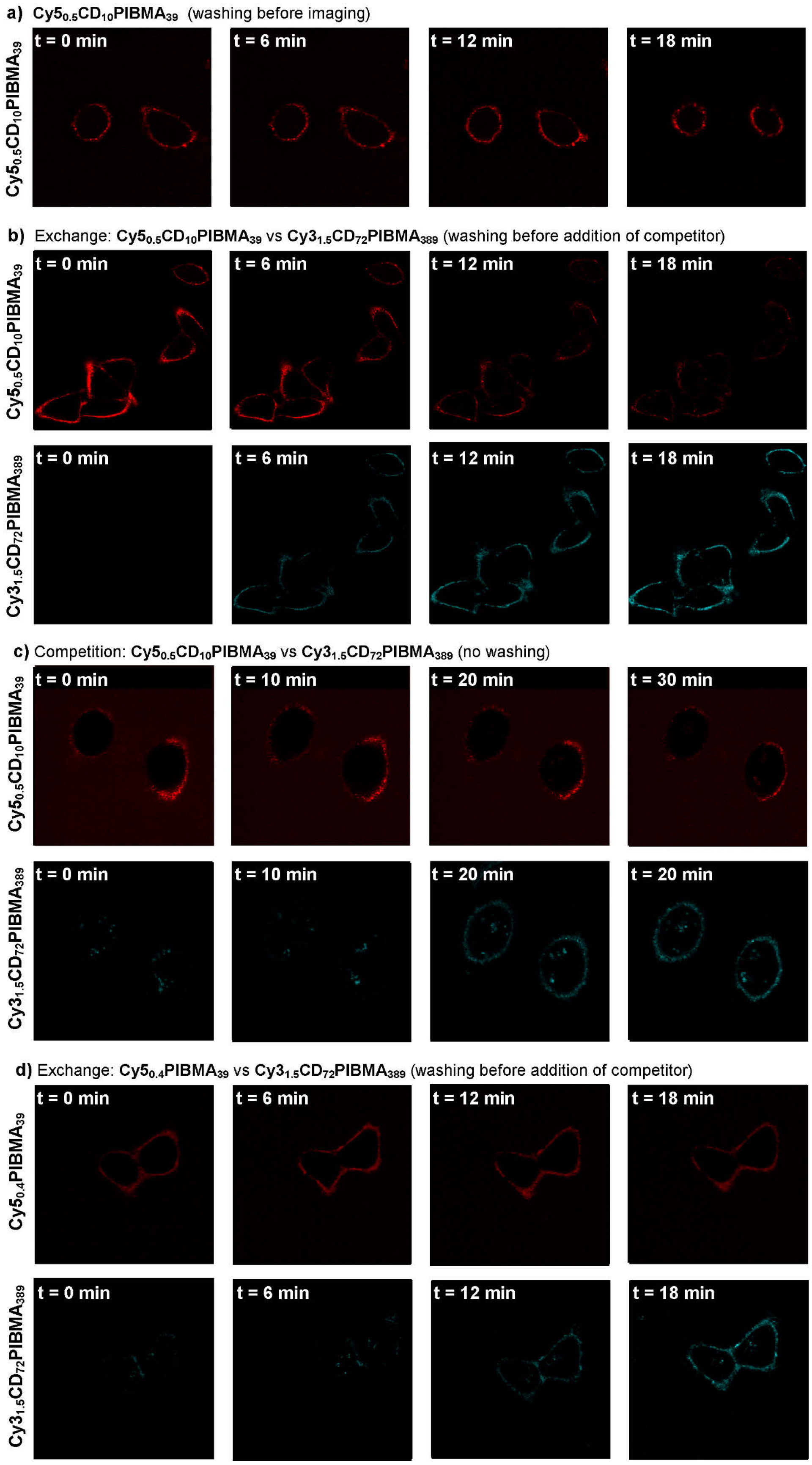

a) $Cy5_{0.5}CD_{10}PIBMA_{39}$ (washing before imaging)
$Cy5_{0.5}CD_{10}PIBMA_{39}$
t = 0 min
t = 6 min
t = 12 min
t = 18 min
b) Exchange: $Cy5_{0.5}CD_{10}PIBMA_{39}$ vs $Cy3_{1.5}CD_{72}PIBMA_{389}$ (washing before addition of competitor)
$Cy5_{0.5}CD_{10}PIBMA_{39}$
t = 0 min
t = 6 min
t = 12 min
t = 18 min
$Cy3_{1.5}CD_{72}PIBMA_{389}$
t = 0 min
t = 6 min
t = 12 min
t = 18 min
c) Competition: $Cy5_{0.5}CD_{10}PIBMA_{39}$ vs $Cy3_{1.5}CD_{72}PIBMA_{389}$ (no washing)
$Cy5_{0.5}CD_{10}PIBMA_{39}$
t = 0 min
t = 10 min
t = 20 min
t = 30 min
$Cy3_{1.5}CD_{72}PIBMA_{389}$
t = 0 min
t = 10 min
t = 20 min
t = 20 min
d) Exchange: $Cy5_{0.4}PIBMA_{39}$ vs $Cy3_{1.5}CD_{72}PIBMA_{389}$ (washing before addition of competitor)
$Cy5_{0.4}PIBMA_{39}$
t = 0 min
t = 6 min
t = 12 min
t = 18 min
$Cy3_{1.5}CD_{72}PIBMA_{389}$
t = 0 min
t = 6 min
t = 12 min
t = 18 min

Figure S18. Reduction in Cy5 signal (**$Cy5_{0.5}CD_{10}PIBMA_{39}$** or **$Cy5_{0.4}PIBMA_{39}$**) under various conditions. **Ac-TZ14011-Ad** functionalized MDAMB231 X4 cells were incubated with **$Cy5_{0.5}CD_{10}PIBMA_{39}$** or **$Cy5_{0.4}PIBMA_{39}$** (1 h), subsequently either a competitor or nothing was added and the cells were imaged over time for a duration of 18 minutes. **a**) **$Cy5_{0.5}CD_{10}PIBMA_{39}$** functionalized cells were washed, but no competitor was added. **b**) **$Cy5_{0.5}CD_{10}PIBMA_{39}$** functionalized cells were washed and **$Cy3_{1.5}CD_{72}PIBMA_{389}$** was added. **c**) **$Cy3_{1.5}CD_{72}PIBMA_{389}$** was directly added to **$Cy5_{0.5}CD_{10}PIBMA_{39}$** functionalized cells, with both polymers in solution the exchange took more time and cells were imaged up to 30 min. **d**) **$Cy5_{0.4}PIBMA_{39}$** functionalized cells were washed and **$Cy3_{1.5}CD_{72}PIBMA_{389}$** was added. With Cy5 (**$Cy5_{0.5}CD_{10}PIBMA_{39}$** or **$Cy5_{0.4}PIBMA_{39}$**) in red and Cy3 (**$Cy3_{1.5}CD_{72}PIBMA_{389}$**) in blue.

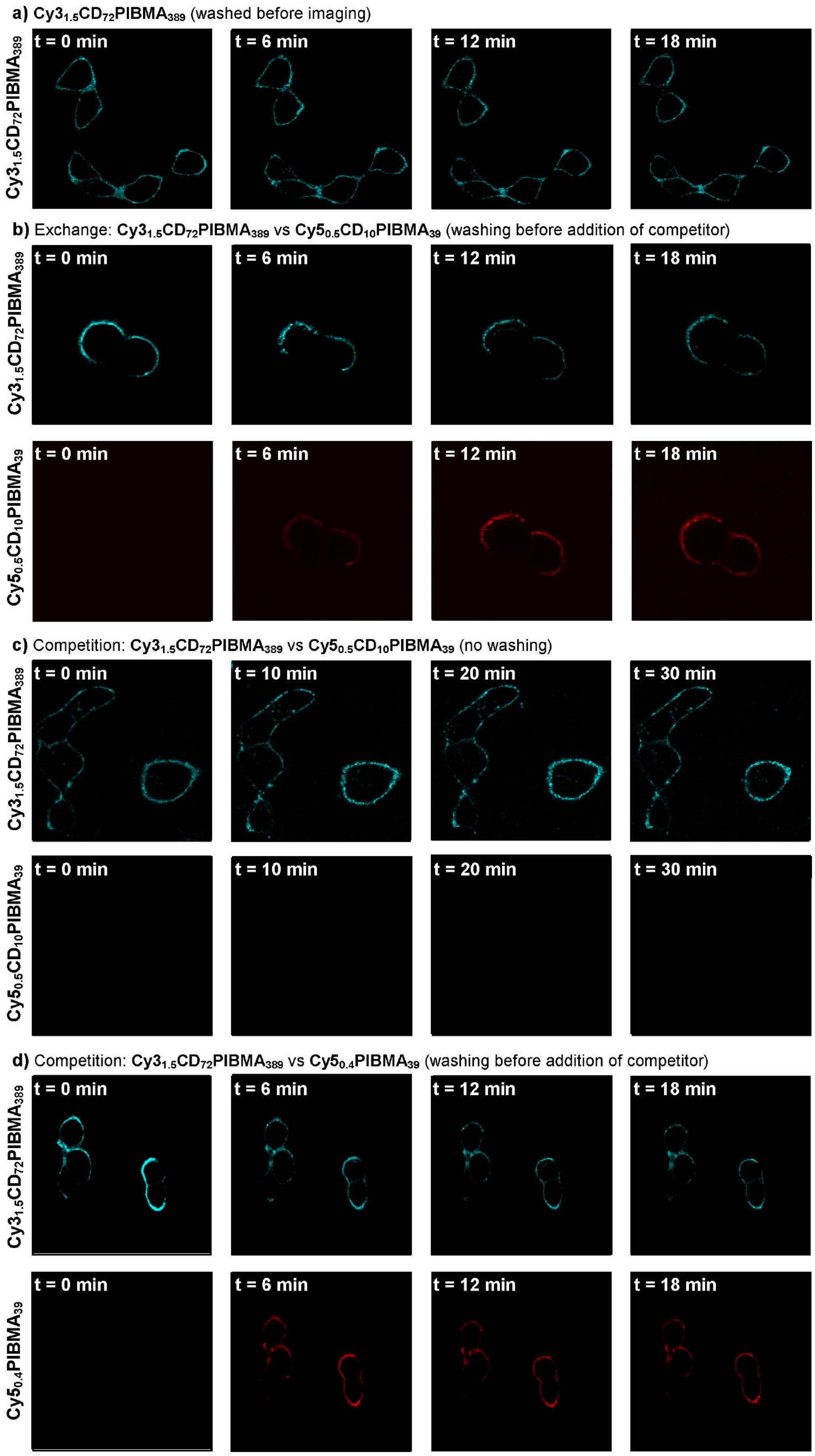
a) $Cy3_{1.5}CD_{72}PIBMA_{389}$ (washed before imaging)
$Cy3_{1.5}CD_{72}PIBMA_{389}$
t = 0 min
t = 6 min
t = 12 min
t = 18 min
b) Exchange: $Cy3_{1.5}CD_{72}PIBMA_{389}$ vs $Cy5_{0.5}CD_{10}PIBMA_{39}$ (washing before addition of competitor)
$Cy3_{1.5}CD_{72}PIBMA_{389}$
$Cy5_{0.5}CD_{10}PIBMA_{39}$
t = 0 min
t = 6 min
t = 12 min
t = 18 min
c) Competition: $Cy3_{1.5}CD_{72}PIBMA_{389}$ vs $Cy5_{0.5}CD_{10}PIBMA_{39}$ (no washing)
$Cy3_{1.5}CD_{72}PIBMA_{389}$
$Cy5_{0.5}CD_{10}PIBMA_{39}$
t = 0 min
t = 10 min
t = 20 min
t = 30 min
d) Competition: $Cy3_{1.5}CD_{72}PIBMA_{389}$ vs $Cy5_{0.4}PIBMA_{39}$ (washing before addition of competitor)
$Cy3_{1.5}CD_{72}PIBMA_{389}$
$Cy5_{0.4}PIBMA_{39}$
t = 0 min
t = 6 min
t = 12 min
t = 18 min

Figure S19. Reduction in Cy3 signal (**$Cy3_{1.5}CD_{72}PIBMA_{389}$**) under various conditions. **Ac-TZ14011-Ad** functionalized MDAMB231 X4 cells were incubated with **$Cy3_{1.5}CD_{72}PIBMA_{389}$** (1 h), subsequently either a competitor or nothing was added and the cells were imaged over time for a duration of 18 minutes. **a**) Cells were washed, but no competitor was added. **b**) Cells were washed and **$Cy5_{0.5}CD_{10}PIBMA_{39}$** was added. **c**) **$Cy5_{0.5}CD_{10}PIBMA_{39}$** was directly added to **$Cy3_{1.5}CD_{72}PIBMA_{389}$** functionalized cells. With both polymers in solution the exchange took more time and cells were imaged up to 30 min. **d**) Cells were washed and **$Cy5_{0.4}PIBMA_{39}$** was added. With Cy5 (**$Cy5_{0.5}CD_{10}PIBMA_{39}$** or **$Cy5_{0.4}PIBMA_{39}$**) in red and Cy3 (**$Cy3_{1.5}CD_{72}PIBMA_{389}$**) in blue.

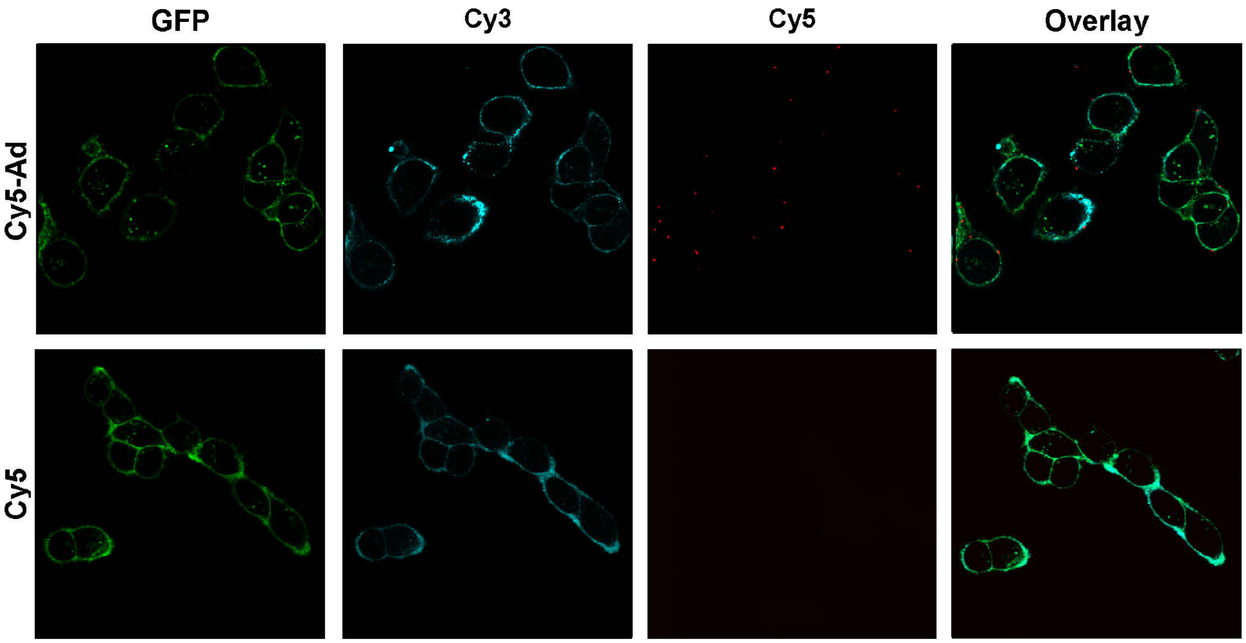

Figure S20: Binding of **Cy5-Ad** or **Cy5** to **$Cy3_{1.5}CD_{72}PIBMA_{389}$** functionalized MDAMB231 X4 cells analyzed by confocal microscopy. For clarity, both the (overlay) image and the same image at the individual channels are displayed, with GFP in green, Cy3 (**$Cy3_{1.5}CD_{72}PIBMA_{389}$**) in blue and Cy5 (**Cy5-Ad** or **Cy5**) in red.

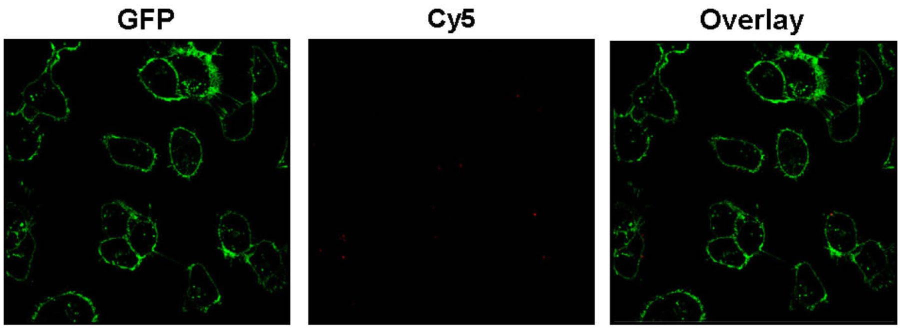

Figure S21. Binding of **Cy5-Ad$_2$** to non-functionalized MDAMB231 X4 cells. **Cy5-Ad$_2$** poorly binds to the cell surfaces when CD$_n$PIBMA$_m$ functionalization is absent. For clarity, both the (overlay) image and the same image at the individual channels are displayed, with GFP in green, and Cy5 (**Cy5-Ad$_2$**) in red.

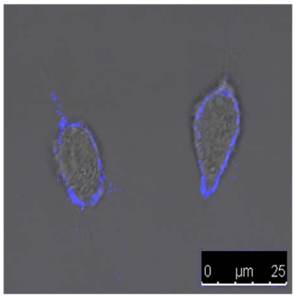

Figure S22: Functionalization of CXCR4 expressing human fetal heart stem cell with **Cy3$_{1.5}$CD$_{72}$PIBMA$_{389}$** (blue) analyzed by confocal microscopy

References

1 Bunschoten, A. *et al.* Tailoring fluorescent dyes to optimize a hybrid RGD-tracer. *Bioconjugate Chem.* **27**, 1253-1258 (2016).

2 Mujumdar, R. B., Ernst, L. A., Mujumdar, S. R., Lewis, C. J. & Waggoner, A. S. Cyanine dye labeling reagents: sulfoindocyanine succinimidyl esters. *Bioconjugate Chem.* **4**, 105-111 (1993).

3 Shershov, V. E. *et al.* Near-infrared heptamethine cyanine dyes. Synthesis, spectroscopic characterization, thermal properties and photostability. *Dyes and Pigments* **97**, 353-360 (2013).

4 Huveneers, S. *et al.* Integrin alpha v beta 3 controls activity and oncogenic potential of primed c-Src. *Cancer Res* **67**, 2693-2700 (2007).

5 Song, J. W. *et al.* Microfluidic endothelium for studying the intravascular adhesion of metastatic breast cancer cells. *PLoS One* **4**, e5756 (2009).

6 van den Berg, N. S., Buckle, T., Kuil, J., Wesseling, J. & van Leeuwen, F. W. B. Immunohistochemical detection of the CXCR4 expression in tumor tissue using the fluorescent peptide antagonist Ac-TZ14011-FITC. *Transl Oncol* **4**, 234-240 (2011).

7 Kuil, J. *et al.* Hybrid peptide dendrimers for imaging of chemokine receptor 4 (CXCR4) expression. *Mol Pharm* **8**, 2444-2453 (2011).

8 Kuil, J. *et al.* Synthesis and Evaluation of a Bimodal CXCR4 Antagonistic Peptide. *Bioconjugate Chemistry* **22**, 859-864 (2011).

9 Mosmann, T. Rapid colorimetric assay for cellular growth and survival: application to proliferation and cytotoxicity assays. *J Immunol Methods* **65**, 55-63 (1983).

10 Gerlier, D. & Thomasset, N. Use of MTT colorimetric assay to measure cell activation. *J Immunol Methods* **94**, 57-63 (1986).

11 Pauwels, E. K. J., Welling, M. M. & Feitsma, R. I. J. The labeling of proteins and LDL with ^{99m}Tc: a new direct method employing KBH_4 and stannous chloride. *Nucl Med Biol* **20**, 825-833 (1993).
